# Measurement of the charge asymmetry in top-quark pair production in the lepton-plus-jets final state in *pp* collision data at $\sqrt{s}=8\,\mathrm TeV{}$ with the ATLAS detector

**DOI:** 10.1140/epjc/s10052-016-3910-6

**Published:** 2016-02-19

**Authors:** Atlas Collaboration, G. Aad, B. Abbott, J. Abdallah, O. Abdinov, R. Aben, M. Abolins, O. S. AbouZeid, H. Abramowicz, H. Abreu, R. Abreu, Y. Abulaiti, B. S. Acharya, L. Adamczyk, D. L. Adams, J. Adelman, S. Adomeit, T. Adye, A. A. Affolder, T. Agatonovic-Jovin, J. Agricola, J. A. Aguilar-Saavedra, S. P. Ahlen, F. Ahmadov, G. Aielli, H. Akerstedt, T. P. A. Åkesson, A. V. Akimov, G. L. Alberghi, J. Albert, S. Albrand, M. J. Alconada Verzini, M. Aleksa, I. N. Aleksandrov, C. Alexa, G. Alexander, T. Alexopoulos, M. Alhroob, G. Alimonti, L. Alio, J. Alison, S. P. Alkire, B. M. M. Allbrooke, P. P. Allport, A. Aloisio, A. Alonso, F. Alonso, C. Alpigiani, A. Altheimer, B. Alvarez Gonzalez, D. Álvarez Piqueras, M. G. Alviggi, B. T. Amadio, K. Amako, Y. Amaral Coutinho, C. Amelung, D. Amidei, S. P. Amor Dos Santos, A. Amorim, S. Amoroso, N. Amram, G. Amundsen, C. Anastopoulos, L. S. Ancu, N. Andari, T. Andeen, C. F. Anders, G. Anders, J. K. Anders, K. J. Anderson, A. Andreazza, V. Andrei, S. Angelidakis, I. Angelozzi, P. Anger, A. Angerami, F. Anghinolfi, A. V. Anisenkov, N. Anjos, A. Annovi, M. Antonelli, A. Antonov, J. Antos, F. Anulli, M. Aoki, L. Aperio Bella, G. Arabidze, Y. Arai, J. P. Araque, A. T. H. Arce, F. A. Arduh, J.-F. Arguin, S. Argyropoulos, M. Arik, A. J. Armbruster, O. Arnaez, H. Arnold, M. Arratia, O. Arslan, A. Artamonov, G. Artoni, S. Asai, N. Asbah, A. Ashkenazi, B. Åsman, L. Asquith, K. Assamagan, R. Astalos, M. Atkinson, N. B. Atlay, K. Augsten, M. Aurousseau, G. Avolio, B. Axen, M. K. Ayoub, G. Azuelos, M. A. Baak, A. E. Baas, M. J. Baca, C. Bacci, H. Bachacou, K. Bachas, M. Backes, M. Backhaus, P. Bagiacchi, P. Bagnaia, Y. Bai, T. Bain, J. T. Baines, O. K. Baker, E. M. Baldin, P. Balek, T. Balestri, F. Balli, W. K. Balunas, E. Banas, Sw. Banerjee, A. A. E. Bannoura, L. Barak, E. L. Barberio, D. Barberis, M. Barbero, T. Barillari, M. Barisonzi, T. Barklow, N. Barlow, S. L. Barnes, B. M. Barnett, R. M. Barnett, Z. Barnovska, A. Baroncelli, G. Barone, A. J. Barr, F. Barreiro, J. Barreiro Guimarães da Costa, R. Bartoldus, A. E. Barton, P. Bartos, A. Basalaev, A. Bassalat, A. Basye, R. L. Bates, S. J. Batista, J. R. Batley, M. Battaglia, M. Bauce, F. Bauer, H. S. Bawa, J. B. Beacham, M. D. Beattie, T. Beau, P. H. Beauchemin, R. Beccherle, P. Bechtle, H. P. Beck, K. Becker, M. Becker, M. Beckingham, C. Becot, A. J. Beddall, A. Beddall, V. A. Bednyakov, C. P. Bee, L. J. Beemster, T. A. Beermann, M. Begel, J. K. Behr, C. Belanger-Champagne, W. H. Bell, G. Bella, L. Bellagamba, A. Bellerive, M. Bellomo, K. Belotskiy, O. Beltramello, O. Benary, D. Benchekroun, M. Bender, K. Bendtz, N. Benekos, Y. Benhammou, E. Benhar Noccioli, J. A. Benitez Garcia, D. P. Benjamin, J. R. Bensinger, S. Bentvelsen, L. Beresford, M. Beretta, D. Berge, E. Bergeaas Kuutmann, N. Berger, F. Berghaus, J. Beringer, C. Bernard, N. R. Bernard, C. Bernius, F. U. Bernlochner, T. Berry, P. Berta, C. Bertella, G. Bertoli, F. Bertolucci, C. Bertsche, D. Bertsche, M. I. Besana, G. J. Besjes, O. Bessidskaia Bylund, M. Bessner, N. Besson, C. Betancourt, S. Bethke, A. J. Bevan, W. Bhimji, R. M. Bianchi, L. Bianchini, M. Bianco, O. Biebel, D. Biedermann, S. P. Bieniek, N. V. Biesuz, M. Biglietti, J. Bilbao De Mendizabal, H. Bilokon, M. Bindi, S. Binet, A. Bingul, C. Bini, S. Biondi, D. M. Bjergaard, C. W. Black, J. E. Black, K. M. Black, D. Blackburn, R. E. Blair, J.-B. Blanchard, J. E. Blanco, T. Blazek, I. Bloch, C. Blocker, W. Blum, U. Blumenschein, S. Blunier, G. J. Bobbink, V. S. Bobrovnikov, S. S. Bocchetta, A. Bocci, C. Bock, M. Boehler, J. A. Bogaerts, D. Bogavac, A. G. Bogdanchikov, C. Bohm, V. Boisvert, T. Bold, V. Boldea, A. S. Boldyrev, M. Bomben, M. Bona, M. Boonekamp, A. Borisov, G. Borissov, S. Borroni, J. Bortfeldt, V. Bortolotto, K. Bos, D. Boscherini, M. Bosman, J. Boudreau, J. Bouffard, E. V. Bouhova-Thacker, D. Boumediene, C. Bourdarios, N. Bousson, S. K. Boutle, A. Boveia, J. Boyd, I. R. Boyko, I. Bozic, J. Bracinik, A. Brandt, G. Brandt, O. Brandt, U. Bratzler, B. Brau, J. E. Brau, H. M. Braun, W. D. Breaden Madden, K. Brendlinger, A. J. Brennan, L. Brenner, R. Brenner, S. Bressler, T. M. Bristow, D. Britton, D. Britzger, F. M. Brochu, I. Brock, R. Brock, J. Bronner, G. Brooijmans, T. Brooks, W. K. Brooks, J. Brosamer, E. Brost, P. A. Bruckman de Renstrom, D. Bruncko, R. Bruneliere, A. Bruni, G. Bruni, M. Bruschi, N. Bruscino, L. Bryngemark, T. Buanes, Q. Buat, P. Buchholz, A. G. Buckley, S. I. Buda, I. A. Budagov, F. Buehrer, L. Bugge, M. K. Bugge, O. Bulekov, D. Bullock, H. Burckhart, S. Burdin, C. D. Burgard, B. Burghgrave, S. Burke, I. Burmeister, E. Busato, D. Büscher, V. Büscher, P. Bussey, J. M. Butler, A. I. Butt, C. M. Buttar, J. M. Butterworth, P. Butti, W. Buttinger, A. Buzatu, A. R. Buzykaev, S. Cabrera Urbán, D. Caforio, V. M. Cairo, O. Cakir, N. Calace, P. Calafiura, A. Calandri, G. Calderini, P. Calfayan, L. P. Caloba, D. Calvet, S. Calvet, R. Camacho Toro, S. Camarda, P. Camarri, D. Cameron, R. Caminal Armadans, S. Campana, M. Campanelli, A. Campoverde, V. Canale, A. Canepa, M. Cano Bret, J. Cantero, R. Cantrill, T. Cao, M. D. M. Capeans Garrido, I. Caprini, M. Caprini, M. Capua, R. Caputo, R. M. Carbone, R. Cardarelli, F. Cardillo, T. Carli, G. Carlino, L. Carminati, S. Caron, E. Carquin, G. D. Carrillo-Montoya, J. R. Carter, J. Carvalho, D. Casadei, M. P. Casado, M. Casolino, E. Castaneda-Miranda, A. Castelli, V. Castillo Gimenez, N. F. Castro, P. Catastini, A. Catinaccio, J. R. Catmore, A. Cattai, J. Caudron, V. Cavaliere, D. Cavalli, M. Cavalli-Sforza, V. Cavasinni, F. Ceradini, B. C. Cerio, K. Cerny, A. S. Cerqueira, A. Cerri, L. Cerrito, F. Cerutti, M. Cerv, A. Cervelli, S. A. Cetin, A. Chafaq, D. Chakraborty, I. Chalupkova, Y. L. Chan, P. Chang, J. D. Chapman, D. G. Charlton, C. C. Chau, C. A. Chavez Barajas, S. Cheatham, A. Chegwidden, S. Chekanov, S. V. Chekulaev, G. A. Chelkov, M. A. Chelstowska, C. Chen, H. Chen, K. Chen, L. Chen, S. Chen, S. Chen, X. Chen, Y. Chen, H. C. Cheng, Y. Cheng, A. Cheplakov, E. Cheremushkina, R. Cherkaoui El Moursli, V. Chernyatin, E. Cheu, L. Chevalier, V. Chiarella, G. Chiarelli, G. Chiodini, A. S. Chisholm, R. T. Chislett, A. Chitan, M. V. Chizhov, K. Choi, S. Chouridou, B. K. B. Chow, V. Christodoulou, D. Chromek-Burckhart, J. Chudoba, A. J. Chuinard, J. J. Chwastowski, L. Chytka, G. Ciapetti, A. K. Ciftci, D. Cinca, V. Cindro, I. A. Cioara, A. Ciocio, F. Cirotto, Z. H. Citron, M. Ciubancan, A. Clark, B. L. Clark, P. J. Clark, R. N. Clarke, C. Clement, Y. Coadou, M. Cobal, A. Coccaro, J. Cochran, L. Coffey, J. G. Cogan, L. Colasurdo, B. Cole, S. Cole, A. P. Colijn, J. Collot, T. Colombo, G. Compostella, P. Conde Muiño, E. Coniavitis, S. H. Connell, I. A. Connelly, V. Consorti, S. Constantinescu, C. Conta, G. Conti, F. Conventi, M. Cooke, B. D. Cooper, A. M. Cooper-Sarkar, T. Cornelissen, M. Corradi, F. Corriveau, A. Corso-Radu, A. Cortes-Gonzalez, G. Cortiana, G. Costa, M. J. Costa, D. Costanzo, D. Côté, G. Cottin, G. Cowan, B. E. Cox, K. Cranmer, G. Cree, S. Crépé-Renaudin, F. Crescioli, W. A. Cribbs, M. Crispin Ortuzar, M. Cristinziani, V. Croft, G. Crosetti, T. Cuhadar Donszelmann, J. Cummings, M. Curatolo, J. Cúth, C. Cuthbert, H. Czirr, P. Czodrowski, S. D’Auria, M. D’Onofrio, M. J. Da Cunha Sargedas De Sousa, C. Da Via, W. Dabrowski, A. Dafinca, T. Dai, O. Dale, F. Dallaire, C. Dallapiccola, M. Dam, J. R. Dandoy, N. P. Dang, A. C. Daniells, M. Danninger, M. Dano Hoffmann, V. Dao, G. Darbo, S. Darmora, J. Dassoulas, A. Dattagupta, W. Davey, C. David, T. Davidek, E. Davies, M. Davies, P. Davison, Y. Davygora, E. Dawe, I. Dawson, R. K. Daya-Ishmukhametova, K. De, R. de Asmundis, A. De Benedetti, S. De Castro, S. De Cecco, N. De Groot, P. de Jong, H. De la Torre, F. De Lorenzi, D. De Pedis, A. De Salvo, U. De Sanctis, A. De Santo, J. B. De Vivie De Regie, W. J. Dearnaley, R. Debbe, C. Debenedetti, D. V. Dedovich, I. Deigaard, J. Del Peso, T. Del Prete, D. Delgove, F. Deliot, C. M. Delitzsch, M. Deliyergiyev, A. Dell’Acqua, L. Dell’Asta, M. Dell’Orso, M. Della Pietra, D. della Volpe, M. Delmastro, P. A. Delsart, C. Deluca, D. A. DeMarco, S. Demers, M. Demichev, A. Demilly, S. P. Denisov, D. Derendarz, J. E. Derkaoui, F. Derue, P. Dervan, K. Desch, C. Deterre, K. Dette, P. O. Deviveiros, A. Dewhurst, S. Dhaliwal, A. Di Ciaccio, L. Di Ciaccio, A. Di Domenico, C. Di Donato, A. Di Girolamo, B. Di Girolamo, A. Di Mattia, B. Di Micco, R. Di Nardo, A. Di Simone, R. Di Sipio, D. Di Valentino, C. Diaconu, M. Diamond, F. A. Dias, M. A. Diaz, E. B. Diehl, J. Dietrich, S. Diglio, A. Dimitrievska, J. Dingfelder, P. Dita, S. Dita, F. Dittus, F. Djama, T. Djobava, J. I. Djuvsland, M. A. B. do Vale, D. Dobos, M. Dobre, C. Doglioni, T. Dohmae, J. Dolejsi, Z. Dolezal, B. A. Dolgoshein, M. Donadelli, S. Donati, P. Dondero, J. Donini, J. Dopke, A. Doria, M. T. Dova, A. T. Doyle, E. Drechsler, M. Dris, E. Dubreuil, E. Duchovni, G. Duckeck, O. A. Ducu, D. Duda, A. Dudarev, L. Duflot, L. Duguid, M. Dührssen, M. Dunford, H. Duran Yildiz, M. Düren, A. Durglishvili, D. Duschinger, B. Dutta, M. Dyndal, C. Eckardt, K. M. Ecker, R. C. Edgar, W. Edson, N. C. Edwards, W. Ehrenfeld, T. Eifert, G. Eigen, K. Einsweiler, T. Ekelof, M. El Kacimi, M. Ellert, S. Elles, F. Ellinghaus, A. A. Elliot, N. Ellis, J. Elmsheuser, M. Elsing, D. Emeliyanov, Y. Enari, O. C. Endner, M. Endo, J. Erdmann, A. Ereditato, G. Ernis, J. Ernst, M. Ernst, S. Errede, E. Ertel, M. Escalier, H. Esch, C. Escobar, B. Esposito, A. I. Etienvre, E. Etzion, H. Evans, A. Ezhilov, L. Fabbri, G. Facini, R. M. Fakhrutdinov, S. Falciano, R. J. Falla, J. Faltova, Y. Fang, M. Fanti, A. Farbin, A. Farilla, T. Farooque, S. Farrell, S. M. Farrington, P. Farthouat, F. Fassi, P. Fassnacht, D. Fassouliotis, M. Faucci Giannelli, A. Favareto, L. Fayard, O. L. Fedin, W. Fedorko, S. Feigl, L. Feligioni, C. Feng, E. J. Feng, H. Feng, A. B. Fenyuk, L. Feremenga, P. Fernandez Martinez, S. Fernandez Perez, J. Ferrando, A. Ferrari, P. Ferrari, R. Ferrari, D. E. Ferreira de Lima, A. Ferrer, D. Ferrere, C. Ferretti, A. Ferretto Parodi, M. Fiascaris, F. Fiedler, A. Filipčič, M. Filipuzzi, F. Filthaut, M. Fincke-Keeler, K. D. Finelli, M. C. N. Fiolhais, L. Fiorini, A. Firan, A. Fischer, C. Fischer, J. Fischer, W. C. Fisher, N. Flaschel, I. Fleck, P. Fleischmann, G. T. Fletcher, G. Fletcher, R. R. M. Fletcher, T. Flick, A. Floderus, L. R. Flores Castillo, M. J. Flowerdew, A. Formica, A. Forti, D. Fournier, H. Fox, S. Fracchia, P. Francavilla, M. Franchini, D. Francis, L. Franconi, M. Franklin, M. Frate, M. Fraternali, D. Freeborn, S. T. French, F. Friedrich, D. Froidevaux, J. A. Frost, C. Fukunaga, E. Fullana Torregrosa, B. G. Fulsom, T. Fusayasu, J. Fuster, C. Gabaldon, O. Gabizon, A. Gabrielli, A. Gabrielli, G. P. Gach, S. Gadatsch, S. Gadomski, G. Gagliardi, P. Gagnon, C. Galea, B. Galhardo, E. J. Gallas, B. J. Gallop, P. Gallus, G. Galster, K. K. Gan, J. Gao, Y. Gao, Y. S. Gao, F. M. Garay Walls, F. Garberson, C. García, J. E. García Navarro, M. Garcia-Sciveres, R. W. Gardner, N. Garelli, V. Garonne, C. Gatti, A. Gaudiello, G. Gaudio, B. Gaur, L. Gauthier, P. Gauzzi, I. L. Gavrilenko, C. Gay, G. Gaycken, E. N. Gazis, P. Ge, Z. Gecse, C. N. P. Gee, Ch. Geich-Gimbel, M. P. Geisler, C. Gemme, M. H. Genest, S. Gentile, M. George, S. George, D. Gerbaudo, A. Gershon, S. Ghasemi, H. Ghazlane, B. Giacobbe, S. Giagu, V. Giangiobbe, P. Giannetti, B. Gibbard, S. M. Gibson, M. Gignac, M. Gilchriese, T. P. S. Gillam, D. Gillberg, G. Gilles, D. M. Gingrich, N. Giokaris, M. P. Giordani, F. M. Giorgi, F. M. Giorgi, P. F. Giraud, P. Giromini, D. Giugni, C. Giuliani, M. Giulini, B. K. Gjelsten, S. Gkaitatzis, I. Gkialas, E. L. Gkougkousis, L. K. Gladilin, C. Glasman, J. Glatzer, P. C. F. Glaysher, A. Glazov, M. Goblirsch-Kolb, J. R. Goddard, J. Godlewski, S. Goldfarb, T. Golling, D. Golubkov, A. Gomes, R. Gonçalo, J. Goncalves Pinto Firmino Da Costa, L. Gonella, S. González de la Hoz, G. Gonzalez Parra, S. Gonzalez-Sevilla, L. Goossens, P. A. Gorbounov, H. A. Gordon, I. Gorelov, B. Gorini, E. Gorini, A. Gorišek, E. Gornicki, A. T. Goshaw, C. Gössling, M. I. Gostkin, D. Goujdami, A. G. Goussiou, N. Govender, E. Gozani, H. M. X. Grabas, L. Graber, I. Grabowska-Bold, P. O. J. Gradin, P. Grafström, J. Gramling, E. Gramstad, S. Grancagnolo, V. Gratchev, H. M. Gray, E. Graziani, Z. D. Greenwood, C. Grefe, K. Gregersen, I. M. Gregor, P. Grenier, J. Griffiths, A. A. Grillo, K. Grimm, S. Grinstein, Ph. Gris, J.-F. Grivaz, J. P. Grohs, A. Grohsjean, E. Gross, J. Grosse-Knetter, G. C. Grossi, Z. J. Grout, L. Guan, J. Guenther, F. Guescini, D. Guest, O. Gueta, E. Guido, T. Guillemin, S. Guindon, U. Gul, C. Gumpert, J. Guo, Y. Guo, S. Gupta, G. Gustavino, P. Gutierrez, N. G. Gutierrez Ortiz, C. Gutschow, C. Guyot, C. Gwenlan, C. B. Gwilliam, A. Haas, C. Haber, H. K. Hadavand, N. Haddad, P. Haefner, S. Hageböck, Z. Hajduk, H. Hakobyan, M. Haleem, J. Haley, D. Hall, G. Halladjian, G. D. Hallewell, K. Hamacher, P. Hamal, K. Hamano, A. Hamilton, G. N. Hamity, P. G. Hamnett, L. Han, K. Hanagaki, K. Hanawa, M. Hance, B. Haney, P. Hanke, R. Hanna, J. B. Hansen, J. D. Hansen, M. C. Hansen, P. H. Hansen, K. Hara, A. S. Hard, T. Harenberg, F. Hariri, S. Harkusha, R. D. Harrington, P. F. Harrison, F. Hartjes, M. Hasegawa, Y. Hasegawa, A. Hasib, S. Hassani, S. Haug, R. Hauser, L. Hauswald, M. Havranek, C. M. Hawkes, R. J. Hawkings, A. D. Hawkins, T. Hayashi, D. Hayden, C. P. Hays, J. M. Hays, H. S. Hayward, S. J. Haywood, S. J. Head, T. Heck, V. Hedberg, L. Heelan, S. Heim, T. Heim, B. Heinemann, L. Heinrich, J. Hejbal, L. Helary, S. Hellman, D. Hellmich, C. Helsens, J. Henderson, R. C. W. Henderson, Y. Heng, C. Hengler, S. Henkelmann, A. Henrichs, A. M. Henriques Correia, S. Henrot-Versille, G. H. Herbert, Y. Hernández Jiménez, G. Herten, R. Hertenberger, L. Hervas, G. G. Hesketh, N. P. Hessey, J. W. Hetherly, R. Hickling, E. Higón-Rodriguez, E. Hill, J. C. Hill, K. H. Hiller, S. J. Hillier, I. Hinchliffe, E. Hines, R. R. Hinman, M. Hirose, D. Hirschbuehl, J. Hobbs, N. Hod, M. C. Hodgkinson, P. Hodgson, A. Hoecker, M. R. Hoeferkamp, F. Hoenig, M. Hohlfeld, D. Hohn, T. R. Holmes, M. Homann, T. M. Hong, W. H. Hopkins, Y. Horii, A. J. Horton, J-Y. Hostachy, S. Hou, A. Hoummada, J. Howard, J. Howarth, M. Hrabovsky, I. Hristova, J. Hrivnac, T. Hryn’ova, A. Hrynevich, C. Hsu, P. J. Hsu, S.-C. Hsu, D. Hu, Q. Hu, X. Hu, Y. Huang, Z. Hubacek, F. Hubaut, F. Huegging, T. B. Huffman, E. W. Hughes, G. Hughes, M. Huhtinen, T. A. Hülsing, N. Huseynov, J. Huston, J. Huth, G. Iacobucci, G. Iakovidis, I. Ibragimov, L. Iconomidou-Fayard, E. Ideal, Z. Idrissi, P. Iengo, O. Igonkina, T. Iizawa, Y. Ikegami, K. Ikematsu, M. Ikeno, Y. Ilchenko, D. Iliadis, N. Ilic, T. Ince, G. Introzzi, P. Ioannou, M. Iodice, K. Iordanidou, V. Ippolito, A. Irles Quiles, C. Isaksson, M. Ishino, M. Ishitsuka, R. Ishmukhametov, C. Issever, S. Istin, J. M. Iturbe Ponce, R. Iuppa, J. Ivarsson, W. Iwanski, H. Iwasaki, J. M. Izen, V. Izzo, S. Jabbar, B. Jackson, M. Jackson, P. Jackson, M. R. Jaekel, V. Jain, K. Jakobs, S. Jakobsen, T. Jakoubek, J. Jakubek, D. O. Jamin, D. K. Jana, E. Jansen, R. Jansky, J. Janssen, M. Janus, G. Jarlskog, N. Javadov, T. Javůrek, L. Jeanty, J. Jejelava, G.-Y. Jeng, D. Jennens, P. Jenni, J. Jentzsch, C. Jeske, S. Jézéquel, H. Ji, J. Jia, Y. Jiang, S. Jiggins, J. Jimenez Pena, S. Jin, A. Jinaru, O. Jinnouchi, M. D. Joergensen, P. Johansson, K. A. Johns, W. J. Johnson, K. Jon-And, G. Jones, R. W. L. Jones, T. J. Jones, J. Jongmanns, P. M. Jorge, K. D. Joshi, J. Jovicevic, X. Ju, P. Jussel, A. Juste Rozas, M. Kaci, A. Kaczmarska, M. Kado, H. Kagan, M. Kagan, S. J. Kahn, E. Kajomovitz, C. W. Kalderon, S. Kama, A. Kamenshchikov, N. Kanaya, S. Kaneti, V. A. Kantserov, J. Kanzaki, B. Kaplan, L. S. Kaplan, A. Kapliy, D. Kar, K. Karakostas, A. Karamaoun, N. Karastathis, M. J. Kareem, E. Karentzos, M. Karnevskiy, S. N. Karpov, Z. M. Karpova, K. Karthik, V. Kartvelishvili, A. N. Karyukhin, K. Kasahara, L. Kashif, R. D. Kass, A. Kastanas, Y. Kataoka, C. Kato, A. Katre, J. Katzy, K. Kawade, K. Kawagoe, T. Kawamoto, G. Kawamura, S. Kazama, V. F. Kazanin, R. Keeler, R. Kehoe, J. S. Keller, J. J. Kempster, H. Keoshkerian, O. Kepka, B. P. Kerševan, S. Kersten, R. A. Keyes, F. Khalil-zada, H. Khandanyan, A. Khanov, A. G. Kharlamov, T. J. Khoo, V. Khovanskiy, E. Khramov, J. Khubua, S. Kido, H. Y. Kim, S. H. Kim, Y. K. Kim, N. Kimura, O. M. Kind, B. T. King, M. King, S. B. King, J. Kirk, A. E. Kiryunin, T. Kishimoto, D. Kisielewska, F. Kiss, K. Kiuchi, O. Kivernyk, E. Kladiva, M. H. Klein, M. Klein, U. Klein, K. Kleinknecht, P. Klimek, A. Klimentov, R. Klingenberg, J. A. Klinger, T. Klioutchnikova, E.-E. Kluge, P. Kluit, S. Kluth, J. Knapik, E. Kneringer, E. B. F. G. Knoops, A. Knue, A. Kobayashi, D. Kobayashi, T. Kobayashi, M. Kobel, M. Kocian, P. Kodys, T. Koffas, E. Koffeman, L. A. Kogan, S. Kohlmann, Z. Kohout, T. Kohriki, T. Koi, H. Kolanoski, M. Kolb, I. Koletsou, A. A. Komar, Y. Komori, T. Kondo, N. Kondrashova, K. Köneke, A. C. König, T. Kono, R. Konoplich, N. Konstantinidis, R. Kopeliansky, S. Koperny, L. Köpke, A. K. Kopp, K. Korcyl, K. Kordas, A. Korn, A. A. Korol, I. Korolkov, E. V. Korolkova, O. Kortner, S. Kortner, T. Kosek, V. V. Kostyukhin, V. M. Kotov, A. Kotwal, A. Kourkoumeli-Charalampidi, C. Kourkoumelis, V. Kouskoura, A. Koutsman, R. Kowalewski, T. Z. Kowalski, W. Kozanecki, A. S. Kozhin, V. A. Kramarenko, G. Kramberger, D. Krasnopevtsev, M. W. Krasny, A. Krasznahorkay, J. K. Kraus, A. Kravchenko, S. Kreiss, M. Kretz, J. Kretzschmar, K. Kreutzfeldt, P. Krieger, K. Krizka, K. Kroeninger, H. Kroha, J. Kroll, J. Kroseberg, J. Krstic, U. Kruchonak, H. Krüger, N. Krumnack, A. Kruse, M. C. Kruse, M. Kruskal, T. Kubota, H. Kucuk, S. Kuday, S. Kuehn, A. Kugel, F. Kuger, A. Kuhl, T. Kuhl, V. Kukhtin, R. Kukla, Y. Kulchitsky, S. Kuleshov, M. Kuna, T. Kunigo, A. Kupco, H. Kurashige, Y. A. Kurochkin, V. Kus, E. S. Kuwertz, M. Kuze, J. Kvita, T. Kwan, D. Kyriazopoulos, A. La Rosa, J. L. La Rosa Navarro, L. La Rotonda, C. Lacasta, F. Lacava, J. Lacey, H. Lacker, D. Lacour, V. R. Lacuesta, E. Ladygin, R. Lafaye, B. Laforge, T. Lagouri, S. Lai, L. Lambourne, S. Lammers, C. L. Lampen, W. Lampl, E. Lançon, U. Landgraf, M. P. J. Landon, V. S. Lang, J. C. Lange, A. J. Lankford, F. Lanni, K. Lantzsch, A. Lanza, S. Laplace, C. Lapoire, J. F. Laporte, T. Lari, F. Lasagni Manghi, M. Lassnig, P. Laurelli, W. Lavrijsen, A. T. Law, P. Laycock, T. Lazovich, O. Le Dortz, E. Le Guirriec, E. Le Menedeu, M. LeBlanc, T. LeCompte, F. Ledroit-Guillon, C. A. Lee, S. C. Lee, L. Lee, G. Lefebvre, M. Lefebvre, F. Legger, C. Leggett, A. Lehan, G. Lehmann Miotto, X. Lei, W. A. Leight, A. Leisos, A. G. Leister, M. A. L. Leite, R. Leitner, D. Lellouch, B. Lemmer, K. J. C. Leney, T. Lenz, B. Lenzi, R. Leone, S. Leone, C. Leonidopoulos, S. Leontsinis, C. Leroy, C. G. Lester, M. Levchenko, J. Levêque, D. Levin, L. J. Levinson, M. Levy, A. Lewis, A. M. Leyko, M. Leyton, B. Li, H. Li, H. L. Li, L. Li, L. Li, S. Li, X. Li, Y. Li, Z. Liang, H. Liao, B. Liberti, A. Liblong, P. Lichard, K. Lie, J. Liebal, W. Liebig, C. Limbach, A. Limosani, S. C. Lin, T. H. Lin, F. Linde, B. E. Lindquist, J. T. Linnemann, E. Lipeles, A. Lipniacka, M. Lisovyi, T. M. Liss, D. Lissauer, A. Lister, A. M. Litke, B. Liu, D. Liu, H. Liu, J. Liu, J. B. Liu, K. Liu, L. Liu, M. Liu, M. Liu, Y. Liu, M. Livan, A. Lleres, J. Llorente Merino, S. L. Lloyd, F. Lo Sterzo, E. Lobodzinska, P. Loch, W. S. Lockman, F. K. Loebinger, A. E. Loevschall-Jensen, K. M. Loew, A. Loginov, T. Lohse, K. Lohwasser, M. Lokajicek, B. A. Long, J. D. Long, R. E. Long, K. A. Looper, L. Lopes, D. Lopez Mateos, B. Lopez Paredes, I. Lopez Paz, J. Lorenz, N. Lorenzo Martinez, M. Losada, P. J. Lösel, X. Lou, A. Lounis, J. Love, P. A. Love, H. Lu, N. Lu, H. J. Lubatti, C. Luci, A. Lucotte, C. Luedtke, F. Luehring, W. Lukas, L. Luminari, O. Lundberg, B. Lund-Jensen, D. Lynn, R. Lysak, E. Lytken, H. Ma, L. L. Ma, G. Maccarrone, A. Macchiolo, C. M. Macdonald, B. Maček, J. Machado Miguens, D. Macina, D. Madaffari, R. Madar, H. J. Maddocks, W. F. Mader, A. Madsen, J. Maeda, S. Maeland, T. Maeno, A. Maevskiy, E. Magradze, K. Mahboubi, J. Mahlstedt, C. Maiani, C. Maidantchik, A. A. Maier, T. Maier, A. Maio, S. Majewski, Y. Makida, N. Makovec, B. Malaescu, Pa. Malecki, V. P. Maleev, F. Malek, U. Mallik, D. Malon, C. Malone, S. Maltezos, V. M. Malyshev, S. Malyukov, J. Mamuzic, G. Mancini, B. Mandelli, L. Mandelli, I. Mandić, R. Mandrysch, J. Maneira, A. Manfredini, L. Manhaes de Andrade Filho, J. Manjarres Ramos, A. Mann, A. Manousakis-Katsikakis, B. Mansoulie, R. Mantifel, M. Mantoani, L. Mapelli, L. March, G. Marchiori, M. Marcisovsky, C. P. Marino, M. Marjanovic, D. E. Marley, F. Marroquim, S. P. Marsden, Z. Marshall, L. F. Marti, S. Marti-Garcia, B. Martin, T. A. Martin, V. J. Martin, B. Martin dit Latour, M. Martinez, S. Martin-Haugh, V. S. Martoiu, A. C. Martyniuk, M. Marx, F. Marzano, A. Marzin, L. Masetti, T. Mashimo, R. Mashinistov, J. Masik, A. L. Maslennikov, I. Massa, L. Massa, P. Mastrandrea, A. Mastroberardino, T. Masubuchi, P. Mättig, J. Mattmann, J. Maurer, S. J. Maxfield, D. A. Maximov, R. Mazini, S. M. Mazza, G. Mc Goldrick, S. P. Mc Kee, A. McCarn, R. L. McCarthy, T. G. McCarthy, N. A. McCubbin, K. W. McFarlane, J. A. Mcfayden, G. Mchedlidze, S. J. McMahon, R. A. McPherson, M. Medinnis, S. Meehan, S. Mehlhase, A. Mehta, K. Meier, C. Meineck, B. Meirose, B. R. Mellado Garcia, F. Meloni, A. Mengarelli, S. Menke, E. Meoni, K. M. Mercurio, S. Mergelmeyer, P. Mermod, L. Merola, C. Meroni, F. S. Merritt, A. Messina, J. Metcalfe, A. S. Mete, C. Meyer, C. Meyer, J-P. Meyer, J. Meyer, H. Meyer Zu Theenhausen, R. P. Middleton, S. Miglioranzi, L. Mijović, G. Mikenberg, M. Mikestikova, M. Mikuž, M. Milesi, A. Milic, D. W. Miller, C. Mills, A. Milov, D. A. Milstead, A. A. Minaenko, Y. Minami, I. A. Minashvili, A. I. Mincer, B. Mindur, M. Mineev, Y. Ming, L. M. Mir, K. P. Mistry, T. Mitani, J. Mitrevski, V. A. Mitsou, A. Miucci, P. S. Miyagawa, J. U. Mjörnmark, T. Moa, K. Mochizuki, S. Mohapatra, W. Mohr, S. Molander, R. Moles-Valls, R. Monden, K. Mönig, C. Monini, J. Monk, E. Monnier, A. Montalbano, J. Montejo Berlingen, F. Monticelli, S. Monzani, R. W. Moore, N. Morange, D. Moreno, M. Moreno Llácer, P. Morettini, D. Mori, T. Mori, M. Morii, M. Morinaga, V. Morisbak, S. Moritz, A. K. Morley, G. Mornacchi, J. D. Morris, S. S. Mortensen, A. Morton, L. Morvaj, M. Mosidze, J. Moss, K. Motohashi, R. Mount, E. Mountricha, S. V. Mouraviev, E. J. W. Moyse, S. Muanza, R. D. Mudd, F. Mueller, J. Mueller, R. S. P. Mueller, T. Mueller, D. Muenstermann, P. Mullen, G. A. Mullier, J. A. Murillo Quijada, W. J. Murray, H. Musheghyan, E. Musto, A. G. Myagkov, M. Myska, B. P. Nachman, O. Nackenhorst, J. Nadal, K. Nagai, R. Nagai, Y. Nagai, K. Nagano, A. Nagarkar, Y. Nagasaka, K. Nagata, M. Nagel, E. Nagy, A. M. Nairz, Y. Nakahama, K. Nakamura, T. Nakamura, I. Nakano, H. Namasivayam, R. F. Naranjo Garcia, R. Narayan, D. I. Narrias Villar, T. Naumann, G. Navarro, R. Nayyar, H. A. Neal, P. Yu. Nechaeva, T. J. Neep, P. D. Nef, A. Negri, M. Negrini, S. Nektarijevic, C. Nellist, A. Nelson, S. Nemecek, P. Nemethy, A. A. Nepomuceno, M. Nessi, M. S. Neubauer, M. Neumann, R. M. Neves, P. Nevski, P. R. Newman, D. H. Nguyen, R. B. Nickerson, R. Nicolaidou, B. Nicquevert, J. Nielsen, N. Nikiforou, A. Nikiforov, V. Nikolaenko, I. Nikolic-Audit, K. Nikolopoulos, J. K. Nilsen, P. Nilsson, Y. Ninomiya, A. Nisati, R. Nisius, T. Nobe, M. Nomachi, I. Nomidis, T. Nooney, S. Norberg, M. Nordberg, O. Novgorodova, S. Nowak, M. Nozaki, L. Nozka, K. Ntekas, G. Nunes Hanninger, T. Nunnemann, E. Nurse, F. Nuti, B. J. O’Brien, F. O’grady, D. C. O’Neil, V. O’Shea, F. G. Oakham, H. Oberlack, T. Obermann, J. Ocariz, A. Ochi, I. Ochoa, J. P. Ochoa-Ricoux, S. Oda, S. Odaka, H. Ogren, A. Oh, S. H. Oh, C. C. Ohm, H. Ohman, H. Oide, W. Okamura, H. Okawa, Y. Okumura, T. Okuyama, A. Olariu, S. A. Olivares Pino, D. Oliveira Damazio, A. Olszewski, J. Olszowska, A. Onofre, K. Onogi, P. U. E. Onyisi, C. J. Oram, M. J. Oreglia, Y. Oren, D. Orestano, N. Orlando, C. Oropeza Barrera, R. S. Orr, B. Osculati, R. Ospanov, G. Otero y Garzon, H. Otono, M. Ouchrif, F. Ould-Saada, A. Ouraou, K. P. Oussoren, Q. Ouyang, A. Ovcharova, M. Owen, R. E. Owen, V. E. Ozcan, N. Ozturk, K. Pachal, A. Pacheco Pages, C. Padilla Aranda, M. Pagáčová, S. Pagan Griso, E. Paganis, F. Paige, P. Pais, K. Pajchel, G. Palacino, S. Palestini, M. Palka, D. Pallin, A. Palma, Y. B. Pan, E. St. Panagiotopoulou, C. E. Pandini, J. G. Panduro Vazquez, P. Pani, S. Panitkin, D. Pantea, L. Paolozzi, Th. D. Papadopoulou, K. Papageorgiou, A. Paramonov, D. Paredes Hernandez, M. A. Parker, K. A. Parker, F. Parodi, J. A. Parsons, U. Parzefall, E. Pasqualucci, S. Passaggio, F. Pastore, Fr. Pastore, G. Pásztor, S. Pataraia, N. D. Patel, J. R. Pater, T. Pauly, J. Pearce, B. Pearson, L. E. Pedersen, M. Pedersen, S. Pedraza Lopez, R. Pedro, S. V. Peleganchuk, D. Pelikan, O. Penc, C. Peng, H. Peng, B. Penning, J. Penwell, D. V. Perepelitsa, E. Perez Codina, M. T. Pérez García-Estañ, L. Perini, H. Pernegger, S. Perrella, R. Peschke, V. D. Peshekhonov, K. Peters, R. F. Y. Peters, B. A. Petersen, T. C. Petersen, E. Petit, A. Petridis, C. Petridou, P. Petroff, E. Petrolo, F. Petrucci, N. E. Pettersson, R. Pezoa, P. W. Phillips, G. Piacquadio, E. Pianori, A. Picazio, E. Piccaro, M. Piccinini, M. A. Pickering, R. Piegaia, D. T. Pignotti, J. E. Pilcher, A. D. Pilkington, A. W. J. Pin, J. Pina, M. Pinamonti, J. L. Pinfold, A. Pingel, S. Pires, H. Pirumov, M. Pitt, C. Pizio, L. Plazak, M.-A. Pleier, V. Pleskot, E. Plotnikova, P. Plucinski, D. Pluth, R. Poettgen, L. Poggioli, D. Pohl, G. Polesello, A. Poley, A. Policicchio, R. Polifka, A. Polini, C. S. Pollard, V. Polychronakos, K. Pommès, L. Pontecorvo, B. G. Pope, G. A. Popeneciu, D. S. Popovic, A. Poppleton, S. Pospisil, K. Potamianos, I. N. Potrap, C. J. Potter, C. T. Potter, G. Poulard, J. Poveda, V. Pozdnyakov, P. Pralavorio, A. Pranko, S. Prasad, S. Prell, D. Price, L. E. Price, M. Primavera, S. Prince, M. Proissl, K. Prokofiev, F. Prokoshin, E. Protopapadaki, S. Protopopescu, J. Proudfoot, M. Przybycien, E. Ptacek, D. Puddu, E. Pueschel, D. Puldon, M. Purohit, P. Puzo, J. Qian, G. Qin, Y. Qin, A. Quadt, D. R. Quarrie, W. B. Quayle, M. Queitsch-Maitland, D. Quilty, S. Raddum, V. Radeka, V. Radescu, S. K. Radhakrishnan, P. Radloff, P. Rados, F. Ragusa, G. Rahal, S. Rajagopalan, M. Rammensee, C. Rangel-Smith, F. Rauscher, S. Rave, T. Ravenscroft, M. Raymond, A. L. Read, N. P. Readioff, D. M. Rebuzzi, A. Redelbach, G. Redlinger, R. Reece, K. Reeves, L. Rehnisch, J. Reichert, H. Reisin, C. Rembser, H. Ren, A. Renaud, M. Rescigno, S. Resconi, O. L. Rezanova, P. Reznicek, R. Rezvani, R. Richter, S. Richter, E. Richter-Was, O. Ricken, M. Ridel, P. Rieck, C. J. Riegel, J. Rieger, O. Rifki, M. Rijssenbeek, A. Rimoldi, L. Rinaldi, B. Ristić, E. Ritsch, I. Riu, F. Rizatdinova, E. Rizvi, S. H. Robertson, A. Robichaud-Veronneau, D. Robinson, J. E. M. Robinson, A. Robson, C. Roda, S. Roe, O. Røhne, A. Romaniouk, M. Romano, S. M. Romano Saez, E. Romero Adam, N. Rompotis, M. Ronzani, L. Roos, E. Ros, S. Rosati, K. Rosbach, P. Rose, P. L. Rosendahl, O. Rosenthal, V. Rossetti, E. Rossi, L. P. Rossi, J. H. N. Rosten, R. Rosten, M. Rotaru, I. Roth, J. Rothberg, D. Rousseau, C. R. Royon, A. Rozanov, Y. Rozen, X. Ruan, F. Rubbo, I. Rubinskiy, V. I. Rud, C. Rudolph, M. S. Rudolph, F. Rühr, A. Ruiz-Martinez, Z. Rurikova, N. A. Rusakovich, A. Ruschke, H. L. Russell, J. P. Rutherfoord, N. Ruthmann, Y. F. Ryabov, M. Rybar, G. Rybkin, N. C. Ryder, A. F. Saavedra, G. Sabato, S. Sacerdoti, A. Saddique, H. F-W. Sadrozinski, R. Sadykov, F. Safai Tehrani, P. Saha, M. Sahinsoy, M. Saimpert, T. Saito, H. Sakamoto, Y. Sakurai, G. Salamanna, A. Salamon, J. E. Salazar Loyola, M. Saleem, D. Salek, P. H. Sales De Bruin, D. Salihagic, A. Salnikov, J. Salt, D. Salvatore, F. Salvatore, A. Salvucci, A. Salzburger, D. Sammel, D. Sampsonidis, A. Sanchez, J. Sánchez, V. Sanchez Martinez, H. Sandaker, R. L. Sandbach, H. G. Sander, M. P. Sanders, M. Sandhoff, C. Sandoval, R. Sandstroem, D. P. C. Sankey, M. Sannino, A. Sansoni, C. Santoni, R. Santonico, H. Santos, I. Santoyo Castillo, K. Sapp, A. Sapronov, J. G. Saraiva, B. Sarrazin, O. Sasaki, Y. Sasaki, K. Sato, G. Sauvage, E. Sauvan, G. Savage, P. Savard, C. Sawyer, L. Sawyer, J. Saxon, C. Sbarra, A. Sbrizzi, T. Scanlon, D. A. Scannicchio, M. Scarcella, V. Scarfone, J. Schaarschmidt, P. Schacht, D. Schaefer, R. Schaefer, J. Schaeffer, S. Schaepe, S. Schaetzel, U. Schäfer, A. C. Schaffer, D. Schaile, R. D. Schamberger, V. Scharf, V. A. Schegelsky, D. Scheirich, M. Schernau, C. Schiavi, C. Schillo, M. Schioppa, S. Schlenker, K. Schmieden, C. Schmitt, S. Schmitt, S. Schmitt, B. Schneider, Y. J. Schnellbach, U. Schnoor, L. Schoeffel, A. Schoening, B. D. Schoenrock, E. Schopf, A. L. S. Schorlemmer, M. Schott, D. Schouten, J. Schovancova, S. Schramm, M. Schreyer, N. Schuh, M. J. Schultens, H.-C. Schultz-Coulon, H. Schulz, M. Schumacher, B. A. Schumm, Ph. Schune, C. Schwanenberger, A. Schwartzman, T. A. Schwarz, Ph. Schwegler, H. Schweiger, Ph. Schwemling, R. Schwienhorst, J. Schwindling, T. Schwindt, F. G. Sciacca, E. Scifo, G. Sciolla, F. Scuri, F. Scutti, J. Searcy, G. Sedov, E. Sedykh, P. Seema, S. C. Seidel, A. Seiden, F. Seifert, J. M. Seixas, G. Sekhniaidze, K. Sekhon, S. J. Sekula, D. M. Seliverstov, N. Semprini-Cesari, C. Serfon, L. Serin, L. Serkin, T. Serre, M. Sessa, R. Seuster, H. Severini, T. Sfiligoj, F. Sforza, A. Sfyrla, E. Shabalina, M. Shamim, L. Y. Shan, R. Shang, J. T. Shank, M. Shapiro, P. B. Shatalov, K. Shaw, S. M. Shaw, A. Shcherbakova, C. Y. Shehu, P. Sherwood, L. Shi, S. Shimizu, C. O. Shimmin, M. Shimojima, M. Shiyakova, A. Shmeleva, D. Shoaleh Saadi, M. J. Shochet, S. Shojaii, S. Shrestha, E. Shulga, M. A. Shupe, S. Shushkevich, P. Sicho, P. E. Sidebo, O. Sidiropoulou, D. Sidorov, A. Sidoti, F. Siegert, Dj. Sijacki, J. Silva, Y. Silver, S. B. Silverstein, V. Simak, O. Simard, Lj. Simic, S. Simion, E. Simioni, B. Simmons, D. Simon, P. Sinervo, N. B. Sinev, M. Sioli, G. Siragusa, A. N. Sisakyan, S. Yu. Sivoklokov, J. Sjölin, T. B. Sjursen, M. B. Skinner, H. P. Skottowe, P. Skubic, M. Slater, T. Slavicek, M. Slawinska, K. Sliwa, V. Smakhtin, B. H. Smart, L. Smestad, S. Yu. Smirnov, Y. Smirnov, L. N. Smirnova, O. Smirnova, M. N. K. Smith, R. W. Smith, M. Smizanska, K. Smolek, A. A. Snesarev, G. Snidero, S. Snyder, R. Sobie, F. Socher, A. Soffer, D. A. Soh, G. Sokhrannyi, C. A. Solans, M. Solar, J. Solc, E. Yu. Soldatov, U. Soldevila, A. A. Solodkov, A. Soloshenko, O. V. Solovyanov, V. Solovyev, P. Sommer, H. Y. Song, N. Soni, A. Sood, A. Sopczak, B. Sopko, V. Sopko, V. Sorin, D. Sosa, M. Sosebee, C. L. Sotiropoulou, R. Soualah, A. M. Soukharev, D. South, B. C. Sowden, S. Spagnolo, M. Spalla, M. Spangenberg, F. Spanò, W. R. Spearman, D. Sperlich, F. Spettel, R. Spighi, G. Spigo, L. A. Spiller, M. Spousta, R. D. St. Denis, A. Stabile, S. Staerz, J. Stahlman, R. Stamen, S. Stamm, E. Stanecka, C. Stanescu, M. Stanescu-Bellu, M. M. Stanitzki, S. Stapnes, E. A. Starchenko, J. Stark, P. Staroba, P. Starovoitov, R. Staszewski, P. Steinberg, B. Stelzer, H. J. Stelzer, O. Stelzer-Chilton, H. Stenzel, G. A. Stewart, J. A. Stillings, M. C. Stockton, M. Stoebe, G. Stoicea, P. Stolte, S. Stonjek, A. R. Stradling, A. Straessner, M. E. Stramaglia, J. Strandberg, S. Strandberg, A. Strandlie, E. Strauss, M. Strauss, P. Strizenec, R. Ströhmer, D. M. Strom, R. Stroynowski, A. Strubig, S. A. Stucci, B. Stugu, N. A. Styles, D. Su, J. Su, R. Subramaniam, A. Succurro, Y. Sugaya, M. Suk, V. V. Sulin, S. Sultansoy, T. Sumida, S. Sun, X. Sun, J. E. Sundermann, K. Suruliz, G. Susinno, M. R. Sutton, S. Suzuki, M. Svatos, M. Swiatlowski, I. Sykora, T. Sykora, D. Ta, C. Taccini, K. Tackmann, J. Taenzer, A. Taffard, R. Tafirout, N. Taiblum, H. Takai, R. Takashima, H. Takeda, T. Takeshita, Y. Takubo, M. Talby, A. A. Talyshev, J. Y. C. Tam, K. G. Tan, J. Tanaka, R. Tanaka, S. Tanaka, B. B. Tannenwald, N. Tannoury, S. Tapia Araya, S. Tapprogge, S. Tarem, F. Tarrade, G. F. Tartarelli, P. Tas, M. Tasevsky, T. Tashiro, E. Tassi, A. Tavares Delgado, Y. Tayalati, F. E. Taylor, G. N. Taylor, P. T. E. Taylor, W. Taylor, F. A. Teischinger, M. Teixeira Dias Castanheira, P. Teixeira-Dias, K. K. Temming, D. Temple, H. Ten Kate, P. K. Teng, J. J. Teoh, F. Tepel, S. Terada, K. Terashi, J. Terron, S. Terzo, M. Testa, R. J. Teuscher, T. Theveneaux-Pelzer, J. P. Thomas, J. Thomas-Wilsker, E. N. Thompson, P. D. Thompson, R. J. Thompson, A. S. Thompson, L. A. Thomsen, E. Thomson, M. Thomson, R. P. Thun, M. J. Tibbetts, R. E. Ticse Torres, V. O. Tikhomirov, Yu. A. Tikhonov, S. Timoshenko, E. Tiouchichine, P. Tipton, S. Tisserant, K. Todome, T. Todorov, S. Todorova-Nova, J. Tojo, S. Tokár, K. Tokushuku, K. Tollefson, E. Tolley, L. Tomlinson, M. Tomoto, L. Tompkins, K. Toms, E. Torrence, H. Torres, E. Torró Pastor, J. Toth, F. Touchard, D. R. Tovey, T. Trefzger, L. Tremblet, A. Tricoli, I. M. Trigger, S. Trincaz-Duvoid, M. F. Tripiana, W. Trischuk, B. Trocmé, C. Troncon, M. Trottier-McDonald, M. Trovatelli, L. Truong, M. Trzebinski, A. Trzupek, C. Tsarouchas, J. C-L. Tseng, P. V. Tsiareshka, D. Tsionou, G. Tsipolitis, N. Tsirintanis, S. Tsiskaridze, V. Tsiskaridze, E. G. Tskhadadze, K. M. Tsui, I. I. Tsukerman, V. Tsulaia, S. Tsuno, D. Tsybychev, A. Tudorache, V. Tudorache, A. N. Tuna, S. A. Tupputi, S. Turchikhin, D. Turecek, R. Turra, A. J. Turvey, P. M. Tuts, A. Tykhonov, M. Tylmad, M. Tyndel, I. Ueda, R. Ueno, M. Ughetto, M. Ugland, F. Ukegawa, G. Unal, A. Undrus, G. Unel, F. C. Ungaro, Y. Unno, C. Unverdorben, J. Urban, P. Urquijo, P. Urrejola, G. Usai, A. Usanova, L. Vacavant, V. Vacek, B. Vachon, C. Valderanis, N. Valencic, S. Valentinetti, A. Valero, L. Valery, S. Valkar, S. Vallecorsa, J. A. Valls Ferrer, W. Van Den Wollenberg, P. C. Van Der Deijl, R. van der Geer, H. van der Graaf, N. van Eldik, P. van Gemmeren, J. Van Nieuwkoop, I. van Vulpen, M. C. van Woerden, M. Vanadia, W. Vandelli, R. Vanguri, A. Vaniachine, F. Vannucci, G. Vardanyan, R. Vari, E. W. Varnes, T. Varol, D. Varouchas, A. Vartapetian, K. E. Varvell, F. Vazeille, T. Vazquez Schroeder, J. Veatch, L. M. Veloce, F. Veloso, T. Velz, S. Veneziano, A. Ventura, D. Ventura, M. Venturi, N. Venturi, A. Venturini, V. Vercesi, M. Verducci, W. Verkerke, J. C. Vermeulen, A. Vest, M. C. Vetterli, O. Viazlo, I. Vichou, T. Vickey, O. E. Vickey Boeriu, G. H. A. Viehhauser, S. Viel, R. Vigne, M. Villa, M. Villaplana Perez, E. Vilucchi, M. G. Vincter, V. B. Vinogradov, I. Vivarelli, F. Vives Vaque, S. Vlachos, D. Vladoiu, M. Vlasak, M. Vogel, P. Vokac, G. Volpi, M. Volpi, H. von der Schmitt, H. von Radziewski, E. von Toerne, V. Vorobel, K. Vorobev, M. Vos, R. Voss, J. H. Vossebeld, N. Vranjes, M. Vranjes Milosavljevic, V. Vrba, M. Vreeswijk, R. Vuillermet, I. Vukotic, Z. Vykydal, P. Wagner, W. Wagner, H. Wahlberg, S. Wahrmund, J. Wakabayashi, J. Walder, R. Walker, W. Walkowiak, C. Wang, F. Wang, H. Wang, H. Wang, J. Wang, J. Wang, K. Wang, R. Wang, S. M. Wang, T. Wang, T. Wang, X. Wang, C. Wanotayaroj, A. Warburton, C. P. Ward, D. R. Wardrope, A. Washbrook, C. Wasicki, P. M. Watkins, A. T. Watson, I. J. Watson, M. F. Watson, G. Watts, S. Watts, B. M. Waugh, S. Webb, M. S. Weber, S. W. Weber, J. S. Webster, A. R. Weidberg, B. Weinert, J. Weingarten, C. Weiser, H. Weits, P. S. Wells, T. Wenaus, T. Wengler, S. Wenig, N. Wermes, M. Werner, P. Werner, M. Wessels, J. Wetter, K. Whalen, A. M. Wharton, A. White, M. J. White, R. White, S. White, D. Whiteson, F. J. Wickens, W. Wiedenmann, M. Wielers, P. Wienemann, C. Wiglesworth, L. A. M. Wiik-Fuchs, A. Wildauer, H. G. Wilkens, H. H. Williams, S. Williams, C. Willis, S. Willocq, A. Wilson, J. A. Wilson, I. Wingerter-Seez, F. Winklmeier, B. T. Winter, M. Wittgen, J. Wittkowski, S. J. Wollstadt, M. W. Wolter, H. Wolters, B. K. Wosiek, J. Wotschack, M. J. Woudstra, K. W. Wozniak, M. Wu, M. Wu, S. L. Wu, X. Wu, Y. Wu, T. R. Wyatt, B. M. Wynne, S. Xella, D. Xu, L. Xu, B. Yabsley, S. Yacoob, R. Yakabe, M. Yamada, D. Yamaguchi, Y. Yamaguchi, A. Yamamoto, S. Yamamoto, T. Yamanaka, K. Yamauchi, Y. Yamazaki, Z. Yan, H. Yang, H. Yang, Y. Yang, W-M. Yao, Y. C. Yap, Y. Yasu, E. Yatsenko, K. H. Yau Wong, J. Ye, S. Ye, I. Yeletskikh, A. L. Yen, E. Yildirim, K. Yorita, R. Yoshida, K. Yoshihara, C. Young, C. J. S. Young, S. Youssef, D. R. Yu, J. Yu, J. M. Yu, J. Yu, L. Yuan, S. P. Y. Yuen, A. Yurkewicz, I. Yusuff, B. Zabinski, R. Zaidan, A. M. Zaitsev, J. Zalieckas, A. Zaman, S. Zambito, L. Zanello, D. Zanzi, C. Zeitnitz, M. Zeman, A. Zemla, Q. Zeng, K. Zengel, O. Zenin, T. Ženiš, D. Zerwas, D. Zhang, F. Zhang, G. Zhang, H. Zhang, J. Zhang, L. Zhang, R. Zhang, X. Zhang, Z. Zhang, X. Zhao, Y. Zhao, Z. Zhao, A. Zhemchugov, J. Zhong, B. Zhou, C. Zhou, L. Zhou, L. Zhou, M. Zhou, N. Zhou, C. G. Zhu, H. Zhu, J. Zhu, Y. Zhu, X. Zhuang, K. Zhukov, A. Zibell, D. Zieminska, N. I. Zimine, C. Zimmermann, S. Zimmermann, Z. Zinonos, M. Zinser, M. Ziolkowski, L. Živković, G. Zobernig, A. Zoccoli, M. zur Nedden, G. Zurzolo, L. Zwalinski

**Affiliations:** 1 Department of Physics, University of Adelaide, Adelaide, Australia; 2 Physics Department, SUNY Albany, Albany, NY USA; 3 Department of Physics, University of Alberta, Edmonton, AB Canada; 4 Department of Physics, Ankara University, Ankara, Turkey; 5 Istanbul Aydin University, Istanbul, Turkey; 6 Division of Physics, TOBB University of Economics and Technology, Ankara, Turkey; 7 LAPP, CNRS/IN2P3 and Université Savoie Mont Blanc, Annecy-le-Vieux, France; 8 High Energy Physics Division, Argonne National Laboratory, Argonne, IL USA; 9 Department of Physics, University of Arizona, Tucson, AZ USA; 10 Department of Physics, The University of Texas at Arlington, Arlington, TX USA; 11 Physics Department, University of Athens, Athens, Greece; 12 Physics Department, National Technical University of Athens, Zografou, Greece; 13 Institute of Physics, Azerbaijan Academy of Sciences, Baku, Azerbaijan; 14 Institut de Física d’Altes Energies and Departament de Física de la Universitat Autònoma de Barcelona, Barcelona, Spain; 15 Institute of Physics, University of Belgrade, Belgrade, Serbia; 16 Department for Physics and Technology, University of Bergen, Bergen, Norway; 17 Physics Division, Lawrence Berkeley National Laboratory and University of California, Berkeley, CA USA; 18 Department of Physics, Humboldt University, Berlin, Germany; 19 Albert Einstein Center for Fundamental Physics and Laboratory for High Energy Physics, University of Bern, Bern, Switzerland; 20 School of Physics and Astronomy, University of Birmingham, Birmingham, UK; 21 Department of Physics, Bogazici University, Istanbul, Turkey; 22 Department of Physics Engineering, Gaziantep University, Gaziantep, Turkey; 23 Department of Physics, Dogus University, Gaziantep, Turkey; 24 INFN Sezione di Bologna, Bologna, Italy; 25 Dipartimento di Fisica e Astronomia, Università di Bologna, Bologna, Italy; 26 Physikalisches Institut, University of Bonn, Bonn, Germany; 27 Department of Physics, Boston University, Boston, MA USA; 28 Department of Physics, Brandeis University, Waltham, MA USA; 29 Universidade Federal do Rio De Janeiro COPPE/EE/IF, Rio de Janeiro, Brazil; 30 Electrical Circuits Department, Federal University of Juiz de Fora (UFJF), Juiz de Fora, Brazil; 31 Federal University of Sao Joao del Rei (UFSJ), Sao Joao del Rei, Brazil; 32 Instituto de Fisica, Universidade de Sao Paulo, São Paulo, Brazil; 33 Physics Department, Brookhaven National Laboratory, Upton, NY USA; 34 Transilvania University of Brasov, Brasov, Romania; 35 National Institute of Physics and Nuclear Engineering, Bucharest, Romania; 36 Physics Department, National Institute for Research and Development of Isotopic and Molecular Technologies, Cluj Napoca, Romania; 37 University Politehnica Bucharest, Bucharest, Romania; 38 West University in Timisoara, Timisoara, Romania; 39 Departamento de Física, Universidad de Buenos Aires, Buenos Aires, Argentina; 40 Cavendish Laboratory, University of Cambridge, Cambridge, UK; 41 Department of Physics, Carleton University, Ottawa, ON Canada; 42 CERN, Geneva, Switzerland; 43 Enrico Fermi Institute, University of Chicago, Chicago, IL USA; 44 Departamento de Física, Pontificia Universidad Católica de Chile, Santiago, Chile; 45 Departamento de Física, Universidad Técnica Federico Santa María, Valparaiso, Chile; 46 Institute of High Energy Physics, Chinese Academy of Sciences, Beijing, China; 47 Department of Modern Physics, University of Science and Technology of China, Hefei, Anhui China; 48 Department of Physics, Nanjing University, Nanjing, Jiangsu China; 49 School of Physics, Shandong University, Shandong, China; 50 Shanghai Key Laboratory for Particle Physics and Cosmology, Department of Physics and Astronomy, Shanghai Jiao Tong University, Shanghai, China; 51 Physics Department, Tsinghua University, Beijing, 100084 China; 52 Laboratoire de Physique Corpusculaire, Clermont Université and Université Blaise Pascal and CNRS/IN2P3, Clermont-Ferrand, France; 53 Nevis Laboratory, Columbia University, Irvington, NY USA; 54 Niels Bohr Institute, University of Copenhagen, Copenhagen, Denmark; 55 INFN Gruppo Collegato di Cosenza, Laboratori Nazionali di Frascati, Frascati, Italy; 56 Dipartimento di Fisica, Università della Calabria, Rende, Italy; 57 AGH University of Science and Technology, Faculty of Physics and Applied Computer Science, Kraków, Poland; 58 Marian Smoluchowski Institute of Physics, Jagiellonian University, Kraków, Poland; 59 Institute of Nuclear Physics, Polish Academy of Sciences, Kraków, Poland; 60 Physics Department, Southern Methodist University, Dallas, TX USA; 61 Physics Department, University of Texas at Dallas, Richardson, TX USA; 62 DESY, Hamburg, Zeuthen, Germany; 63 Institut für Experimentelle Physik IV, Technische Universität Dortmund, Dortmund, Germany; 64 Institut für Kern- und Teilchenphysik, Technische Universität Dresden, Dresden, Germany; 65 Department of Physics, Duke University, Durham, NC USA; 66 SUPA-School of Physics and Astronomy, University of Edinburgh, Edinburgh, UK; 67 INFN Laboratori Nazionali di Frascati, Frascati, Italy; 68 Fakultät für Mathematik und Physik, Albert-Ludwigs-Universität, Freiburg, Germany; 69 Section de Physique, Université de Genève, Geneva, Switzerland; 70 INFN Sezione di Genova, Genoa, Italy; 71 Dipartimento di Fisica, Università di Genova, Genoa, Italy; 72 E. Andronikashvili Institute of Physics, Iv. Javakhishvili Tbilisi State University, Tbilisi, Georgia; 73 High Energy Physics Institute, Tbilisi State University, Tbilisi, Georgia; 74 II Physikalisches Institut, Justus-Liebig-Universität Giessen, Giessen, Germany; 75 SUPA-School of Physics and Astronomy, University of Glasgow, Glasgow, UK; 76 II Physikalisches Institut, Georg-August-Universität, Göttingen, Germany; 77 Laboratoire de Physique Subatomique et de Cosmologie, Université Grenoble-Alpes, CNRS/IN2P3, Grenoble, France; 78 Department of Physics, Hampton University, Hampton, VA USA; 79 Laboratory for Particle Physics and Cosmology, Harvard University, Cambridge, MA USA; 80 Kirchhoff-Institut für Physik, Ruprecht-Karls-Universität Heidelberg, Heidelberg, Germany; 81 Physikalisches Institut, Ruprecht-Karls-Universität Heidelberg, Heidelberg, Germany; 82 ZITI Institut für technische Informatik, Ruprecht-Karls-Universität Heidelberg, Mannheim, Germany; 83 Faculty of Applied Information Science, Hiroshima Institute of Technology, Hiroshima, Japan; 84 Department of Physics, The Chinese University of Hong Kong, Shatin, N.T. Hong Kong; 85 Department of Physics, The University of Hong Kong, Pokfulam, Hong Kong; 86 Department of Physics, The Hong Kong University of Science and Technology, Clear Water Bay, Kowloon, Hong Kong, China; 87 Department of Physics, Indiana University, Bloomington, IN USA; 88 Institut für Astro- und Teilchenphysik, Leopold-Franzens-Universität, Innsbruck, Austria; 89 University of Iowa, Iowa City, IA USA; 90 Department of Physics and Astronomy, Iowa State University, Ames, IA USA; 91 Joint Institute for Nuclear Research, JINR Dubna, Dubna, Russia; 92 KEK, High Energy Accelerator Research Organization, Tsukuba, Japan; 93 Graduate School of Science, Kobe University, Kobe, Japan; 94 Faculty of Science, Kyoto University, Kyoto, Japan; 95 Kyoto University of Education, Kyoto, Japan; 96 Department of Physics, Kyushu University, Fukuoka, Japan; 97 Instituto de Física La Plata, Universidad Nacional de La Plata and CONICET, La Plata, Argentina; 98 Physics Department, Lancaster University, Lancaster, UK; 99 INFN Sezione di Lecce, Lecce, Italy; 100 Dipartimento di Matematica e Fisica, Università del Salento, Lecce, Italy; 101 Oliver Lodge Laboratory, University of Liverpool, Liverpool, UK; 102 Department of Physics, Jožef Stefan Institute and University of Ljubljana, Ljubljana, Slovenia; 103 School of Physics and Astronomy, Queen Mary University of London, London, UK; 104 Department of Physics, Royal Holloway University of London, Surrey, UK; 105 Department of Physics and Astronomy, University College London, London, UK; 106 Louisiana Tech University, Ruston, LA USA; 107 Laboratoire de Physique Nucléaire et de Hautes Energies, UPMC and Université Paris-Diderot and CNRS/IN2P3, Paris, France; 108 Fysiska institutionen, Lunds universitet, Lund, Sweden; 109 Departamento de Fisica Teorica C-15, Universidad Autonoma de Madrid, Madrid, Spain; 110 Institut für Physik, Universität Mainz, Mainz, Germany; 111 School of Physics and Astronomy, University of Manchester, Manchester, UK; 112 CPPM, Aix-Marseille Université and CNRS/IN2P3, Marseille, France; 113 Department of Physics, University of Massachusetts, Amherst, MA USA; 114 Department of Physics, McGill University, Montreal, QC Canada; 115 School of Physics, University of Melbourne, Melbourne, VIC Australia; 116 Department of Physics, The University of Michigan, Ann Arbor, MI USA; 117 Department of Physics and Astronomy, Michigan State University, East Lansing, MI USA; 118 INFN Sezione di Milano, Milan, Italy; 119 Dipartimento di Fisica, Università di Milano, Milan, Italy; 120 B.I. Stepanov Institute of Physics, National Academy of Sciences of Belarus, Minsk, Republic of Belarus; 121 National Scientific and Educational Centre for Particle and High Energy Physics, Minsk, Republic of Belarus; 122 Department of Physics, Massachusetts Institute of Technology, Cambridge, MA USA; 123 Group of Particle Physics, University of Montreal, Montreal, QC Canada; 124 P.N. Lebedev Institute of Physics, Academy of Sciences, Moscow, Russia; 125 Institute for Theoretical and Experimental Physics (ITEP), Moscow, Russia; 126 National Research Nuclear University MEPhI, Moscow, Russia; 127 D.V. Skobeltsyn Institute of Nuclear Physics, M.V. Lomonosov Moscow State University, Moscow, Russia; 128 Fakultät für Physik, Ludwig-Maximilians-Universität München, Munich, Germany; 129 Max-Planck-Institut für Physik (Werner-Heisenberg-Institut), Munich, Germany; 130 Nagasaki Institute of Applied Science, Nagasaki, Japan; 131 Graduate School of Science and Kobayashi-Maskawa Institute, Nagoya University, Nagoya, Japan; 132 INFN Sezione di Napoli, Naples, Italy; 133 Dipartimento di Fisica, Università di Napoli, Naples, Italy; 134 Department of Physics and Astronomy, University of New Mexico, Albuquerque, NM USA; 135 Institute for Mathematics, Astrophysics and Particle Physics, Radboud University Nijmegen/Nikhef, Nijmegen, The Netherlands; 136 Nikhef National Institute for Subatomic Physics and University of Amsterdam, Amsterdam, The Netherlands; 137 Department of Physics, Northern Illinois University, De Kalb, IL USA; 138 Budker Institute of Nuclear Physics, SB RAS, Novosibirsk, Russia; 139 Department of Physics, New York University, New York, NY USA; 140 Ohio State University, Columbus, OH USA; 141 Faculty of Science, Okayama University, Okayama, Japan; 142 Homer L. Dodge Department of Physics and Astronomy, University of Oklahoma, Norman, OK USA; 143 Department of Physics, Oklahoma State University, Stillwater, OK USA; 144 Palacký University, RCPTM, Olomouc, Czech Republic; 145 Center for High Energy Physics, University of Oregon, Eugene, OR USA; 146 LAL, Université Paris-Sud and CNRS/IN2P3, Orsay, France; 147 Graduate School of Science, Osaka University, Osaka, Japan; 148 Department of Physics, University of Oslo, Oslo, Norway; 149 Department of Physics, Oxford University, Oxford, UK; 150 INFN Sezione di Pavia, Pavia, Italy; 151 Dipartimento di Fisica, Università di Pavia, Pavia, Italy; 152 Department of Physics, University of Pennsylvania, Philadelphia, PA USA; 153 National Research Centre “Kurchatov Institute” B.P. Konstantinov, Petersburg Nuclear Physics Institute, St. Petersburg, Russia; 154 INFN Sezione di Pisa, Pisa, Italy; 155 Dipartimento di Fisica E. Fermi, Università di Pisa, Pisa, Italy; 156 Department of Physics and Astronomy, University of Pittsburgh, Pittsburgh, PA USA; 157 Laboratório de Instrumentação e Física Experimental de Partículas-LIP, Lisbon, Portugal; 158 Faculdade de Ciências, Universidade de Lisboa, Lisbon, Portugal; 159 Department of Physics, University of Coimbra, Coimbra, Portugal; 160 Centro de Física Nuclear da Universidade de Lisboa, Lisbon, Portugal; 161 Departamento de Fisica, Universidade do Minho, Braga, Portugal; 162 Departamento de Fisica Teorica y del Cosmos and CAFPE, Universidad de Granada, Granada, Spain; 163 Dep Fisica and CEFITEC of Faculdade de Ciencias e Tecnologia, Universidade Nova de Lisboa, Caparica, Portugal; 164 Institute of Physics, Academy of Sciences of the Czech Republic, Prague, Czech Republic; 165 Czech Technical University in Prague, Prague, Czech Republic; 166 Faculty of Mathematics and Physics, Charles University in Prague, Prague, Czech Republic; 167 State Research Center Institute for High Energy Physics, Protvino, Russia; 168 Particle Physics Department, Rutherford Appleton Laboratory, Didcot, UK; 169 INFN Sezione di Roma, Rome, Italy; 170 Dipartimento di Fisica, Sapienza Università di Roma, Rome, Italy; 171 INFN Sezione di Roma Tor Vergata, Rome, Italy; 172 Dipartimento di Fisica, Università di Roma Tor Vergata, Rome, Italy; 173 INFN Sezione di Roma Tre, Rome, Italy; 174 Dipartimento di Matematica e Fisica, Università Roma Tre, Rome, Italy; 175 Faculté des Sciences Ain Chock, Réseau Universitaire de Physique des Hautes Energies-Université Hassan II, Casablanca, Morocco; 176 Centre National de l’Energie des Sciences Techniques Nucleaires, Rabat, Morocco; 177 Faculté des Sciences Semlalia, Université Cadi Ayyad, LPHEA-Marrakech, Marrakech, Morocco; 178 Faculté des Sciences, Université Mohamed Premier and LPTPM, Oujda, Morocco; 179 Faculté des Sciences, Université Mohammed V, Rabat, Morocco; 180 DSM/IRFU (Institut de Recherches sur les Lois Fondamentales de l’Univers), CEA Saclay (Commissariat à l’Energie Atomique et aux Energies Alternatives), Gif-sur-Yvette, France; 181 Santa Cruz Institute for Particle Physics, University of California Santa Cruz, Santa Cruz, CA USA; 182 Department of Physics, University of Washington, Seattle, WA USA; 183 Department of Physics and Astronomy, University of Sheffield, Sheffield, UK; 184 Department of Physics, Shinshu University, Nagano, Japan; 185 Fachbereich Physik, Universität Siegen, Siegen, Germany; 186 Department of Physics, Simon Fraser University, Burnaby, BC Canada; 187 SLAC National Accelerator Laboratory, Stanford, CA USA; 188 Faculty of Mathematics, Physics and Informatics, Comenius University, Bratislava, Slovak Republic; 189 Department of Subnuclear Physics, Institute of Experimental Physics of the Slovak Academy of Sciences, Kosice, Slovak Republic; 190 Department of Physics, University of Cape Town, Cape Town, South Africa; 191 Department of Physics, University of Johannesburg, Johannesburg, South Africa; 192 School of Physics, University of the Witwatersrand, Johannesburg, South Africa; 193 Department of Physics, Stockholm University, Stockholm, Sweden; 194 The Oskar Klein Centre, Stockholm, Sweden; 195 Physics Department, Royal Institute of Technology, Stockholm, Sweden; 196 Departments of Physics and Astronomy and Chemistry, Stony Brook University, Stony Brook, NY USA; 197 Department of Physics and Astronomy, University of Sussex, Brighton, UK; 198 School of Physics, University of Sydney, Sydney, Australia; 199 Institute of Physics, Academia Sinica, Taipei, Taiwan; 200 Department of Physics, Technion: Israel Institute of Technology, Haifa, Israel; 201 Raymond and Beverly Sackler School of Physics and Astronomy, Tel Aviv University, Tel Aviv, Israel; 202 Department of Physics, Aristotle University of Thessaloniki, Thessaloníki, Greece; 203 International Center for Elementary Particle Physics and Department of Physics, The University of Tokyo, Tokyo, Japan; 204 Graduate School of Science and Technology, Tokyo Metropolitan University, Tokyo, Japan; 205 Department of Physics, Tokyo Institute of Technology, Tokyo, Japan; 206 Department of Physics, University of Toronto, Toronto, ON Canada; 207 TRIUMF, Vancouver, BC Canada; 208 Department of Physics and Astronomy, York University, Toronto, ON Canada; 209 Faculty of Pure and Applied Sciences, and Center for Integrated Research in Fundamental Science and Engineering, University of Tsukuba, Tsukuba, Japan; 210 Department of Physics and Astronomy, Tufts University, Medford, MA USA; 211 Centro de Investigaciones, Universidad Antonio Narino, Bogotá, Colombia; 212 Department of Physics and Astronomy, University of California Irvine, Irvine, CA USA; 213 INFN Gruppo Collegato di Udine, Sezione di Trieste, Udine, Italy; 214 ICTP, Trieste, Italy; 215 Dipartimento di Chimica Fisica e Ambiente, Università di Udine, Udine, Italy; 216 Department of Physics, University of Illinois, Urbana, IL USA; 217 Department of Physics and Astronomy, University of Uppsala, Uppsala, Sweden; 218 Instituto de Física Corpuscular (IFIC) and Departamento de Física Atómica, Molecular y Nuclear and Departamento de Ingeniería Electrónica and Instituto de Microelectrónica de Barcelona (IMB-CNM), University of Valencia and CSIC, Valencia, Spain; 219 Department of Physics, University of British Columbia, Vancouver, BC Canada; 220 Department of Physics and Astronomy, University of Victoria, Victoria, BC Canada; 221 Department of Physics, University of Warwick, Coventry, UK; 222 Waseda University, Tokyo, Japan; 223 Department of Particle Physics, The Weizmann Institute of Science, Rehovot, Israel; 224 Department of Physics, University of Wisconsin, Madison, WI USA; 225 Fakultät für Physik und Astronomie, Julius-Maximilians-Universität, Würzburg, Germany; 226 Fachbereich C Physik, Bergische Universität Wuppertal, Wuppertal, Germany; 227 Department of Physics, Yale University, New Haven, CT USA; 228 Yerevan Physics Institute, Yerevan, Armenia; 229 Centre de Calcul de l’Institut National de Physique Nucléaire et de Physique des Particules (IN2P3), Villeurbanne, France

## Abstract

This paper reports inclusive and differential measurements of the $t\bar{t}$ charge asymmetry $A_{\text {C}}$ in $20.3~{\mathrm{fb}^{-1}}$ of $\sqrt{s} = 8~\mathrm TeV{}$ $pp$ collisions recorded by the ATLAS experiment at the Large Hadron Collider at CERN. Three differential measurements are performed as a function of the invariant mass, transverse momentum and longitudinal boost of the $t\bar{t}$ system. The $t\bar{t}$ pairs are selected in the single-lepton channels (*e* or $\mu$) with at least four jets, and a likelihood fit is used to reconstruct the $t\bar{t}$ event kinematics. A Bayesian unfolding procedure is performed to infer the asymmetry at parton level from the observed data distribution. The inclusive $t\bar{t}$ charge asymmetry is measured to be $A_{\text {C}}{} = 0.009 \pm 0.005$ (stat. $+$ syst.). The inclusive and differential measurements are compatible with the values predicted by the Standard Model.

## Introduction

The 8 TeV proton–proton (*pp*) collision data delivered by the CERN Large Hadron Collider (LHC) represents a unique laboratory for precision measurements of the top-quark properties. One interesting feature of $t\bar{t}$ production is the difference in rapidity between top quarks and top antiquarks. In *pp* collisions, this distinct behaviour of top quarks and antiquarks is called the charge asymmetry, $A_{\text {C}}$ [defined in Eq. (1) below]. The Standard Model (SM) expectation computed at next-to-leading order (NLO) in quantum chromodynamics (QCD), including electroweak corrections, predicts $A_{\text {C}}$ to be at the one percent level [1]. Previous asymmetry measurements at the LHC by both the CMS and ATLAS collaborations based on the 7 TeV data, and by the CMS collaboration based on the 8 TeV data, do not report any significant deviation from the SM predictions [2–7]. Charge asymmetry measurements are largely limited by the size of the available data sample, and therefore the larger dataset recorded by the ATLAS detector at $\sqrt{s}$ = 8 TeV allows for an improvement on the precision of the measurement from the $\sqrt{s}$= 7 TeV dataset.

At hadron colliders, $t\bar{t}$ production is predicted to be symmetric under the exchange of top quark and antiquark at leading order (LO). At NLO, the process $q\bar{q}\rightarrow t\bar{t}g$ develops an asymmetry in the top-quark rapidity distributions, due to interference between processes with initial- and final-state gluon emission. The interference between the Born and the NLO diagrams of the $q\bar{q}\rightarrow t\bar{t}$ process also produces an asymmetry. The $qg\rightarrow t\bar{t} {}g$ production process is also asymmetric, but its contribution is much smaller than that from $q\bar{q}$ .

In $q\bar{q}$ scattering processes in $p\bar{p}$ collisions at the Tevatron, the direction of the incoming quark almost always coincides with that of the proton, and this knowledge of the direction of the incoming quarks allows one to define a direct measurement of the forward-backward asymmetry, $A_{\mathrm {FB}}$ [8–11]. In *pp* collisions at the LHC, since the colliding beams are symmetric, it is not possible to use the direction of the incoming quark to define an asymmetry. However, valence quarks carry on average a larger fraction of the proton momentum than sea antiquarks, hence top quarks are more forward and top antiquarks are more central. Using this feature it is possible to define a forward–central asymmetry for the $t\bar{t}$ production, referred to as the charge asymmetry, $A_{\text {C}}$ [8, 12, 13] :

1 $$\begin{aligned} A_{\text {C}}{} = \frac{N(\Delta {}|y|{}>0) - N(\Delta {}|y|{} <0)}{N(\Delta {}|y|{} >0) + N(\Delta {}|y|{} <0)}, \end{aligned}$$

where $\Delta {}|y|{} \equiv |y_t| - |y_{\bar{t}}|$ is the difference between the absolute value of the top-quark rapidity $|y_t|$ and the absolute value of the top-antiquark rapidity $|y_{\bar{t}}|$. At the LHC, the dominant mechanism for $t\bar{t}$ production is the gluon fusion process, while production via the $q\bar{q}$ or the *qg* interactions is small. Since ${gg \rightarrow t\bar{t}}$ processes are charge-symmetric, they only contribute to the denominator of Eq. (1), thus diluting the asymmetry.

Several processes beyond the Standard Model (BSM) can alter $A_{\text {C}}$ [12, 14–25], either with anomalous vector or axial-vector couplings (e.g. axigluons) or via interference with SM processes. Different models also predict different asymmetries as a function of the invariant mass $m_{t\bar{t} {}}$, the transverse momentum $p_{\text {T},t\bar{t} {}}$ and the longitudinal boost $\beta _{z,t\bar{t} {}}$ along the *z*-axis^1^ of the $t\bar{t}$ system [26]. The interest in precisely measuring charge asymmetries in top-quark pair production at the LHC has grown after the CDF and D0 collaborations reported measurements of $A_{\mathrm {FB}}$ that were significantly larger than the SM predictions, in both the inclusive and differential case as a function of $m_{t\bar{t} {}}$ and of the rapidity of the $t\bar{t}$ system, $y_{t\bar{t} {}}$ [10, 11, 27–30]. For the most general BSM scenarios [31], the $A_{\text {C}}$ measurements from the LHC are still compatible with the Tevatron results. However, for specific simple models [20], tension still exists between the LHC and Tevatron results. This motivates the interest in a more precise measurement of the $t\bar{t}$ production charge asymmetry at the LHC.

In this paper, a measurement of the $t\bar{t}$ production charge asymmetry in the single-lepton final state is reported. To allow for comparisons with theory calculations, a Bayesian unfolding procedure is applied to account for distortions due to the acceptance and detector effects, leading to parton-level $A_{\text {C}}$ measurements. The data sample at a centre-of-mass energy of 8 TeV, corresponding to an integrated luminosity of $20.3~\text{ fb }^{-1}$ [32], is used to measure $A_{\text {C}}$ inclusively and differentially as a function of $m_{t\bar{t} {}}$, $p_{\text {T},t\bar{t} {}}$ and $\beta _{z,t\bar{t} {}}$.

This paper is organised as follows. The ATLAS detector is introduced in Sect. 2, followed by the object reconstruction in Sect. 3 and the event selection in Sect. 4. The signal and background modelling is described in Sect. 5 and the procedure to measure $A_{\text {C}}{}$ in Sect. 6. Finally, the results are presented and interpreted in Sect. 7, followed by the conclusions in Sect. 8.

## ATLAS detector

The ATLAS detector [33] consists of the following main subsystems: an inner tracking system immersed in a 2 T magnetic field provided by a superconducting solenoid, electromagnetic (EM) and hadronic calorimeters, and a muon spectrometer incorporating three large superconducting toroid magnets composed of eight coils each. The inner detector (ID) is composed of three subsystems: the pixel detector, the semiconductor tracker and the transition radiation tracker. The ID provides tracking information in the pseudorapidity^2^ range $|\eta |<2.5$, calorimeters measure energy deposits (clusters) for $|\eta |<4.9$, and the muon spectrometer records tracks within $|\eta |<2.7$. A three-level trigger system [34] is used to select interesting events. It consists of a level-1 hardware trigger, reducing the event rate to at most 75 kHz, followed by two software-based trigger levels, collectively referred to as the high-level trigger, yielding a recorded event rate of approximately 400 Hz on average, depending on the data-taking conditions.

## Object reconstruction

This measurement makes use of reconstructed electrons, muons, jets, *b*-jets and missing transverse momentum. A brief summary of the main reconstruction and identification criteria applied for each of these objects is given below.

Electron candidates are reconstructed from clusters in the EM calorimeter that are matched to reconstructed tracks in the inner detector. They are required to have a transverse energy, $E_{\text {T}}$, greater than $25~\mathrm GeV{}$ and $|\eta _{\mathrm cluster}| < 2.47$, where $\eta _{\mathrm cluster}$ is the pseudorapidity of the electromagnetic energy cluster in the calorimeter with respect to the geometric centre of the detector. Candidates are required to satisfy the *tight* quality requirements [35] and are excluded if reconstructed in the transition region between the barrel and endcap sections of the EM calorimeter, $1.37 < |\eta _{\mathrm cluster}| < 1.52$. They are also required to originate less than 2 mm along the *z*-axis (longitudinal impact parameter) from the selected event primary vertex (PV)^3^ and to satisfy two isolation criteria. The first one is calorimeter-based and consists of a requirement on the transverse energy sum of cells within a cone of size $\Delta R = 0.2$ around the electron direction. The second one is a track-based isolation requirement made on the track transverse momentum ($p_{\text {T}}$) sum around the electron in a cone of size $\Delta R = 0.3$. In both cases, the contribution from the electron itself is excluded and the isolation cuts are optimised to individually result in a 90 % efficiency for prompt electrons from $Z\rightarrow e^+e^-$ decays.

Muon candidates [36, 37] are reconstructed using the combined information from the muon spectrometer and the inner detector. They are required to satisfy $p_{\text {T}} > 25~\mathrm GeV{}$ and $|\eta |<2.5$ and analogously to electrons, the muon track longitudinal impact parameter with respect to the PV is required to be less than 2 mm. Muons are required to satisfy a $p_{\text {T}}$-dependent track-based isolation: the scalar sum of the track $p_{\text {T}}$ within a cone of variable size around the muon, $\Delta R =10\,\mathrm GeV{}/p_{\text {T}} ^\mu$ (excluding the muon track itself) must be less than 5 % of the muon $p_{\text {T}}$ ($p_{\text {T}} ^\mu$), corresponding to a 97 % selection efficiency for prompt muons from $Z\rightarrow \mu ^+\mu ^-$ decays.

Jets are reconstructed with the anti-$k_t$ algorithm [38–40] with a radius parameter $R=0.4$ from calibrated topological clusters [33] built from energy deposits in the calorimeters. Prior to jet finding, a local cluster calibration scheme [41, 42] is applied to correct the topological cluster energies for the effects of the noncompensating response of the calorimeter, dead material and out-of-cluster leakage. The corrections are obtained from simulations of charged and neutral particles and validated with data. After energy calibration [43], jets are required to have $p_{\text {T}} > 25~\mathrm GeV{}$ and $|\eta | < 2.5$. Jets from additional simultaneous *pp* interactions (pileup) are suppressed by requiring that the absolute value of the jet vertex fraction (JVF)^4^ for candidates with $p_{\text {T}} <50~\mathrm GeV{}$ and $|\eta |<2.4$ is above 0.5 [44]. All high-$p_{\text {T}}$ electrons are also reconstructed as jets, so the closest jet within $\Delta R=$ 0.2 of a selected electron is discarded to avoid double counting of electrons as jets. Finally, if selected electrons or muons lie within $\Delta R$ = 0.4 of selected jets, they are discarded.

Jets are identified as originating from the hadronisation of a *b*-quark (*b*-tagged) via an algorithm that uses multivariate techniques to combine information from the impact parameters of displaced tracks as well as topological properties of secondary and tertiary decay vertices reconstructed within the jet [45, 46]. The algorithm’s operating point used for this measurement corresponds to 70 % efficiency to tag *b*-quark jets, a rejection factor for light-quark and gluon jets of $\sim$130 and a rejection factor of $\sim$5 for *c*-quark jets, as determined for jets with $p_{\text {T}} >20~\mathrm GeV$ and $|\eta |<2.5$ in simulated $t\bar{t}$ events.

The missing transverse momentum (with magnitude $E_{\text {T}}^{\text {miss}}$) is constructed from the negative vector sum of all calorimeter energy deposits [47]. The ones contained in topological clusters are calibrated at the energy scale of the associated high-$p_{\text {T}}$ object (e.g. jet or electron). The topological cluster energies are corrected using the local cluster calibration scheme discussed in the jet reconstruction paragraph above. The remaining contributions to the $E_{\text {T}}^{\text {miss}}$ are called unclustered energy. In addition, the $E_{\text {T}}^{\text {miss}}$ calculation includes contributions from the selected muons, and muon energy deposits in the calorimeter are removed to avoid double counting.

## Event selection

Only events recorded with an isolated or non-isolated single-electron or single-muon trigger under stable beam conditions with all detector subsystems operational are considered.

The triggers have thresholds on $p^\ell _{\mathrm T}$, the transverse momentum (energy) of the muon (electron). These thresholds are 24 GeV for isolated single-lepton triggers and 60 (36) GeV for non-isolated single-electron (single-muon) triggers. Events satisfying the trigger selection are required to have at least one reconstructed vertex with at least five associated tracks of $p_{\text {T}} > 400$ MeV, consistent with originating from the beam collision region in the *x*–*y* plane. If more than one vertex is found, the hard-scatter PV is taken to be the one which has the largest sum of the squared transverse momenta of its associated tracks.

Events are required to have exactly one candidate electron or muon and at least four jets satisfying the quality and kinematic criteria discussed in Sect. 3. The selected lepton is required to match, with $\Delta R < 0.15$, the lepton reconstructed by the high-level trigger. Events with additional electrons satisfying a looser identification criteria based on a likelihood variable [48] are rejected in order to suppress di-leptonic backgrounds ($t\bar{t}$ or $Z$+jets). At this point, the events are separated into three signal regions defined by the number of *b*-tagged jets (zero, one and at least two).

In order to further suppress multijet and $Z$+jets backgrounds in events with exactly zero or one *b*-tagged jets, the following requirements on $E_{\text {T}}^{\text {miss}}$ and $m_{\mathrm T}^{W}$ 5 are applied: $m_{\mathrm T}^{W}{} + E_{\text {T}}^{\text {miss}} {} > 60~\mathrm GeV{}$ for events with exactly zero or one *b*-tagged jets, and $E_{\text {T}}^{\text {miss}} {} > 40~(20)~\mathrm GeV{}$ for events with exactly zero (one) *b*-tagged jets.

After the event selection, the main background is the production of *W*+jets events. Small contributions arise from multijet, single top quark, *Z*+jets and diboson (*WW*, *WZ*, *ZZ*) production. For events with exactly one (at least two) *b*-tagged jet(s), 216,465 (193,418) data events are observed, of which 68 % (89 %) are expected to be $t\bar{t}$ .

## Signal and background modelling

Monte Carlo simulated samples are used to model the $t\bar{t}$ signal and all backgrounds except for those from multijet events, which are estimated from data. All simulated samples utilise Photos (version 2.15) [49] to simulate photon radiation and Tauola (version 1.20) [50] to simulate $\tau$ decays. They also include simultaneous *pp* interactions (pile-up), generated using Pythia 8.1 [51], and reweighted to the number of interactions per bunch crossing in data (on average 21 in 2012). Most of them are processed through a full Geant4 [52] simulation of the detector response [53], and only the alternative $t\bar{t}$ samples described in Sect. 5.1 are produced using the ATLAS fast simulation that employs parameterised showers in the calorimeters [54]. Finally, the simulated events are reconstructed using the same software as the data. Further details on the modelling of the signal and each of the backgrounds are provided below.

### $t\bar{t}$ signal

The default simulated $t\bar{t}$ events are generated with the NLO generator Powheg-Box (version 1, r2330) [55–57] using the CT10 PDF set [58] interfaced to Pythia (version 6.427) [59] with the CTEQ6L1 PDF set and the Perugia2011C set of tunable parameters (tune) [60] for the underlying event (UE). The $h_{\mathrm {damp}}$ factor, which is the model parameter that controls matrix element/parton shower matching in Powheg-Box and effectively regulates the high-$p_{\text {T}}$ radiation, is set to the top-quark mass.

The alternative samples used to study the modelling of $t\bar{t}$ are:

- Mc@nlo (version 4.01) [61] using the CT10 PDF set and interfaced to Herwig (version 6.520) [62] and Jimmy (version 4.31) [63].
- Powheg-Box using the CT10 PDF and setting the $h_{\mathrm {damp}}$ parameter to infinity, interfaced to Pythia (version 6.426) with the CTEQ6L1 PDF set and the Perugia2011C UE tune.
- Powheg-Box using the CT10 PDF and setting the $h_{\mathrm {damp}}$ parameter to infinity, and interfaced to Herwig with the CTEQ6L1 PDF set and Jimmy to simulate the UE.
- AcerMC [64] using the CTEQ6L1 PDF set and interfaced to Pythia (version 6.426).

All $t\bar{t}$ samples are generated assuming a top-quark mass of $172.5~\mathrm GeV{}$ and are normalised to the theoretical cross section of $\sigma _{t\bar{t}}= 253^{+13}_{-15}$ pb calculated at next-to-next-to-leading order (NNLO) in QCD including resummation of next-to-next-to-leading logarithmic (NNLL) soft gluon terms with Top++ v2.0 [65–71].

### *W* / *Z*+jets background

Samples of events with a *W* or *Z* boson produced in association with jets (*W* / *Z*+jets) are generated with up to five additional partons using the Alpgen (version 2.14) [72] LO generator and the CTEQ6L1 PDF set, interfaced to Pythia (version 6.426) for parton showering and fragmentation. To avoid double counting of partonic configurations generated by both the matrix-element calculation and the parton shower, a parton–jet matching scheme (“MLM matching”) [73] is employed. The *W*+jets samples are generated separately for *W*+light-jets, $Wb\bar{b}$+jets, $Wc\bar{c}$+jets, and *Wc*+jets. The *Z*+jets samples are generated separately for *Z*+light-jets, $Zb\bar{b}$+jets, and $Zc\bar{c}$+jets. Overlap between $W/ZQ\bar{Q}$+jets ($Q=b,c$) events generated from the matrix-element calculation and those generated from parton-shower evolution in the *W* / *Z*+light-jets samples is avoided via an algorithm based on the angular separation between the extra heavy quarks: if $\Delta R(Q,\bar{Q})>0.4$, the matrix-element prediction is used, otherwise the parton-shower prediction is used. The *Z*+jets background is normalised to its inclusive NNLO theoretical cross section [74], while data is used to normalise *W*+jets (see below for details). Further corrections are applied to *Z*+jets simulated events in order to better describe data in the preselected sample. A correction to the heavy-flavour fraction was derived to reproduce the relative rates of *Z*+2-jets events with zero and one *b*-tagged jets observed in data. In addition, the *Z* boson $p_{\text {T}}$ spectrum was compared between data and the simulation in *Z*+2-jets events, and a reweighting function was derived in order to improve the modelling as described in Ref. [75].

The procedure to estimate the normalisation of the $W$+jets background in data exploits the difference in production cross section at the LHC between $W^+$ and $W^-$, where the $W^+$ production cross section is higher than $W^-$ [76]. This is due to the higher density of *u* quarks in protons with respect to *d* quarks, which causes more $u\bar{d}\rightarrow W^+$ to be produced than $d\bar{u}\rightarrow W^-$. The *W* boson charge asymmetry is then defined as the difference between the numbers of events with a single positive or negative lepton divided by the sum. The prediction for the *W* boson charge asymmetry in $W$+jets production is little affected by theoretical uncertainties and can be exploited, in combination with constraints from $W^+$ and $W^{-}$ data samples, to derive the correct overall normalisation for the MC sample prediction. The *W* boson charge asymmetry depends on the flavour composition of the sample, as the size and sign of the asymmetry varies for $Wb\bar{b}$+jets, $Wc\bar{c}$+jets, *Wc*+jets, and *W*+light-jets production. The in situ calibration procedure embedded in the unfolding and described in Sect. 6.4, uses different signal and control regions to determine the normalisation of the $W$+jets background.

### Multijet background

Multijet events can enter the selected data sample through several production and misreconstruction mechanisms. In the electron channel, the multijet background consists of non-prompt electrons from heavy-flavour decays or photon conversion or jets with a high fraction of their energy deposited in the EM calorimeter. In the muon channel, the background contributed by multijet events is predominantly due to final states with non-prompt muons, such as those from semileptonic *b*- or *c*-hadron decays. The multijet background normalisation and shape are estimated from data using the “Matrix Method” (MM) technique.

The MM exploits differences in the properties used for lepton identification between prompt, isolated leptons from *W* and *Z* boson decays (referred to as “real leptons”) and those where the leptons are either non-isolated or result from the misidentification of photons or jets (referred to as “fake leptons”). For this purpose, two samples are defined after imposing the event selection described in Sect. 4, differing only in the lepton identification criteria: a “tight” sample and a “loose” sample, the former being a subset of the latter. The tight selection employs the final lepton identification criteria used in the analysis. For the loose selection, the lepton isolation requirements are omitted for both the muon and electron channels, and the quality requirements are also loosened for the electron channel. The method assumes that the number of selected events in each sample ($N^{\mathrm {loose}}$ and $N^{\mathrm {tight}}$) can be expressed as a linear combination of the numbers of events with real and fake leptons, so that the number of multijet events in the tight sample is given by

2 $$\begin{aligned} N^{\mathrm {tight}}_{\mathrm {multijet}} = \frac{\epsilon _{\mathrm {fake}}}{\epsilon _{\mathrm {real}}-\epsilon _{\mathrm {fake}}}(\epsilon _{\mathrm {real}}N^{\mathrm {loose}}- N^{\mathrm {tight}}) \end{aligned}$$

where $\epsilon _{\mathrm {real}}$ ($\epsilon _{\mathrm {fake}}$) represents the probability for a real (fake) lepton that satisfies the loose criteria to also satisfy the tight. Both of these probabilities are measured in data control samples. To measure $\epsilon _{\mathrm {real}}$, samples enriched in real leptons from *W* boson decays are selected by requiring high $E_{\text {T}}^{\text {miss}}$ or transverse mass $m_{\mathrm T}^{W}$. The average $\epsilon _{\mathrm {real}}$ is 0.75 (0.98) in the electron (muon) channel. To measure $\epsilon _{\mathrm {fake}}$, samples enriched in multijet background are selected by requiring either low $E_{\text {T}}^{\text {miss}}$ (electron channel) or high transverse impact parameter significance for the lepton track (muon channel). The average $\epsilon _{\mathrm {fake}}$ value is 0.35 (0.20) in the electron (muon) channel. Dependencies of $\epsilon _{\mathrm {real}}$ and $\epsilon _{\mathrm {fake}}$ on quantities such as lepton $p_{\text {T}}$ and $\eta$, $\Delta R$ between the lepton and the closest jet, or number of *b*-tagged jets, are parameterised in order to obtain a more accurate estimate.

### Other backgrounds

Samples of single-top-quark backgrounds corresponding to the *t*-channel, *s*-channel and *Wt* production mechanisms are generated with Powheg-Box (version 3.0) [77, 78] using the CT10 PDF set. All samples are generated assuming a top-quark mass of $172.5~\mathrm GeV{}$ and are interfaced to Pythia (version 6.425) with the CTEQ6L1 PDF set and the Perugia2011C UE tune. Overlaps between the $t\bar{t}$ and *Wt* final states are removed using the “diagram removal” scheme [79]. The single-top-quark samples are normalised to the approximate NNLO theoretical cross sections [80–82] using the MSTW 2008 NNLO PDF set.

Most of the diboson *WW* / *WZ* / *ZZ*+jets samples are generated using Alpgen (version 2.13), with up to three additional partons, and using the CTEQ6L1 PDF set, interfaced to Herwig and Jimmy (version 4.31) for parton showering, fragmentation and UE modelling. For the *WW*+jets samples, it is required that at least one of the *W* bosons decays leptonically, while for the *WZ* / *ZZ*+jets samples, it is demanded that at least one of the *Z* bosons decays leptonically. Additional samples of *WZ*+jets, requiring the *W* and *Z* bosons to decay leptonically and hadronically, respectively, are generated with up to three additional partons, including massive *b*- and *c*-quarks, using Sherpa v1.4.1 [83] and the CT10 PDF set. All diboson samples are normalised to their NLO theoretical cross sections [84].

## Charge asymmetry measurement

To measure the charge asymmetry in top-quark pair events, the full $t\bar{t}$ system is reconstructed (Sect. 6.1) and the $\Delta {}|y|$ spectra are unfolded to measure parton-level charge asymmetries (Sect. 6.2) using the estimation of the backgrounds and systematic uncertainties (Sect. 6.3). Significant improvements to the analysis method with respect to the 7 TeV measurement [4] have been made, and these improvements are detailed in the description of the measurement in Sect. 6.4.

**Fig. 1:**
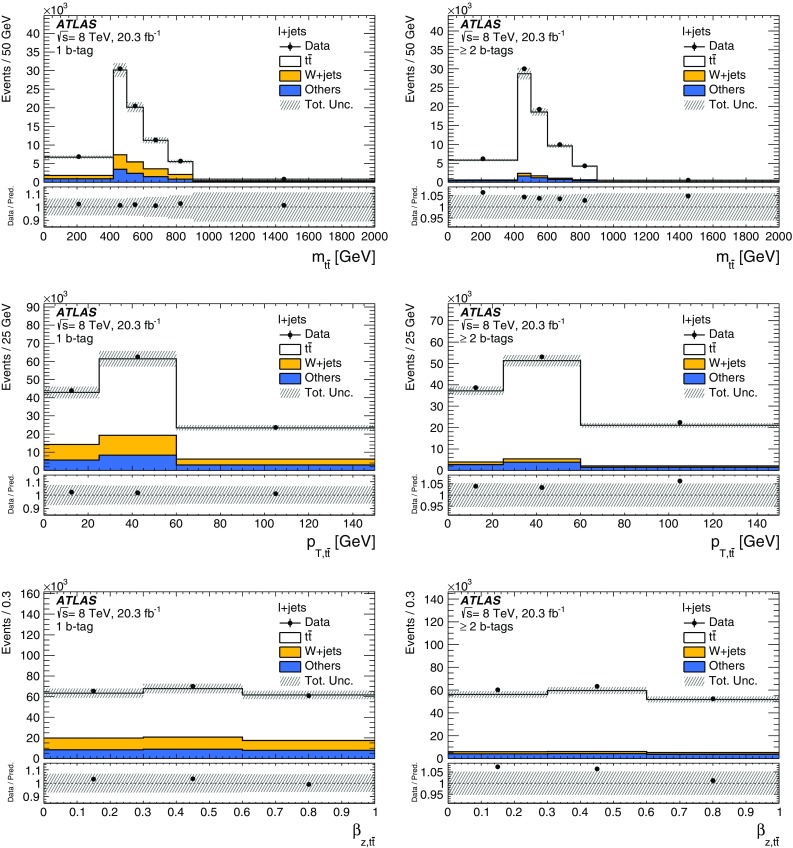
Comparison between data and prediction for the $e$+jets and $\mu {}$+jets channels combined for distributions of kinematic quantities, in the sample with one *b*-tagged jet (*left*) and in the sample with at least two *b*-tagged jets (*right*). *From top to bottom* invariant mass $m_{t\bar{t} {}}$, transverse momentum $p_{\text {T},t\bar{t} {}}$, *z*-component of the velocity of the $t\bar{t}$ system $\beta _{z,t\bar{t} {}}$. The total uncertainty, before the unfolding process, on the signal and background estimation is shown together with statistical uncertainty as a *black hashed band*, and the binnings are those that are used for the differential measurements. The *bottom part of each plot* shows the ratio of the data to the predicted value together with combined statistical and systematic uncertainties

### Reconstruction of the $t\bar{t}$ kinematics

The reconstruction of the $t\bar{t}$ system is achieved using a kinematic fit [85] that assesses the compatibility of the observed event with the decays of a $t\bar{t} {}$ pair based on a likelihood approach. The basic reconstruction method is explained in Ref. [86], but some modifications are introduced as discussed in the following paragraph.

In events with four or five jets, all jets are considered in the fit. For events where more than five jets are reconstructed, only the two jets with the highest likelihood to be *b*-jets, according to the multivariate selection (see Sect. 3), and, of the remaining jets, the three with the highest $p_{\text {T}}$ are considered in the fit. This selection of input jets for the likelihood was chosen to optimise the correct-sign fraction of reconstructed $\Delta {}|y|$. The average correct-sign fraction is estimated with simulation studies and found to be 72 and 75 % in events with exactly one and at least two *b*-tagged jets, respectively. The most probable combination out of all the possible jet permutations is chosen. Permutations with non-*b*-tagged jets assigned as *b*-jets and vice versa have a reduced weight due to the tagging probability in the likelihood. Finally, the lepton charge $Q_\ell$ is used to determine if the reconstructed semileptonically-decaying quark is a top quark ($Q_\ell >0$) or an anti-top quark ($Q_\ell <0$). The distributions of reconstructed quantities, $m_{t\bar{t} {}}$, $p_{\text {T},t\bar{t} {}}$ and $\beta _{z,t\bar{t} {}}$ are shown in Fig. 1, with the binnings that are used in the differential measurements.

### Unfolding

The reconstructed $\Delta {}|y|$ distributions are distorted by acceptance and detector resolution effects. An unfolding procedure is used to estimate the true $\Delta {}|y|$ spectrum, as defined by the *t* and $\bar{t}$ after radiation and before decay in Monte Carlo events, from the one measured in data. The observed spectrum is unfolded using the fully Bayesian unfolding (FBU) technique [87].

The FBU method consists of the strict application of Bayesian inference to the problem of unfolding. This application can be stated in the following terms: given an observed spectrum $\varvec{D}$ with $N_{\mathrm r}$ reconstructed bins, and a response matrix $\mathcal {M}$ with $N_{\mathrm r} \times N_{\mathrm t}$ bins giving the detector response to a true spectrum with $N_{\mathrm t}$ bins, the posterior probability density of the true spectrum $\varvec{T}{}$ (with $N_{\mathrm t}$ bins) follows the probability density

3 $$\begin{aligned}&p\left( \varvec{T}{}|\varvec{D}{}\right) \propto {} \mathcal {L}\left( \varvec{D}{}|\varvec{T}{}\right) \cdot {} \pi {}\left( \varvec{T}{}\right) , \end{aligned}$$

where $\mathcal {L}\left( \varvec{D}{}|\varvec{T}{}\right)$ is the likelihood function of $\varvec{D}$ given $\varvec{T}$ and $\mathcal {M}$, and $\pi {}\left( \varvec{T}{}\right)$ is the prior probability density for $\varvec{T}$. While the response matrix is estimated from the simulated sample of $t\bar{t}$ events, a uniform prior probability density in all bins is chosen as $\pi {}\left( \varvec{T}{}\right)$, such that equal probabilities to all $\varvec{T}$ spectra within a wide range are assigned. The unfolded asymmetry $A_{\text {C}}$ is computed from $p\left( \varvec{T}{}|\varvec{D}{}\right)$ as

4 $$\begin{aligned} p\left( A_{\text {C}}{}|\varvec{D}{}\right) = \int { \delta (A_{\text {C}}{} - A_{\text {C}}{}(\varvec{T}{})) p\left( \varvec{T}{}|\varvec{D}{}\right) \mathrm {d} \varvec{T}{}}. \end{aligned}$$

The treatment of systematic uncertainties is consistently included in the Bayesian inference approach by extending the likelihood $\mathcal {L}\left( \varvec{D}{}|\varvec{T}{}\right)$ with nuisance parameter terms. The marginal likelihood is defined as

5 $$\begin{aligned} \mathcal {L}\left( \varvec{D}{}|\varvec{T}{}\right) = \int \mathcal {L}\left( \varvec{D}{}|\varvec{T}{},\varvec{\theta {}}{}\right) \cdot {}\mathcal {N}(\varvec{\theta {}}{})~\mathrm {d}\varvec{\theta {}}{}, \end{aligned}$$

where $\varvec{\theta {}}$ are the nuisance parameters, and $\mathcal {N}(\varvec{\theta {}}{})$ their prior probability densities, which are assumed to be Normal distributions with mean $\mu =0$ and standard deviation $\sigma =1$. A nuisance parameter is associated with each of the uncertainty sources (as explained below).

The marginalisation approach provides a natural framework to treat simultaneously the unfolding and background estimation using multiple data regions. Given the distributions $\varvec{D}{}_i$ measured in $N_{\text {ch}}$ independent channels, the likelihood is extended to the product of likelihoods of each channel, so that

6 $$\begin{aligned} \mathcal {L}\left( \{\varvec{D}{}_1\cdots {}\varvec{D}{}_{N_{\mathrm ch}}\}|\varvec{T}{}\right) = \int \prod _{i=1}^{N_{\mathrm ch}}\mathcal {L}\left( \varvec{D}{}_i|\varvec{T}{},\varvec{\theta {}}{}\right) \cdot {} \mathcal {N}(\varvec{\theta {}}{}) ~\mathrm {d}\varvec{\theta {}}{}, \end{aligned}$$

where the nuisance parameters are common to all analysis channels.

### Systematic uncertainties

Several sources of systematic uncertainty are considered, which can affect the normalisation of signal and background and/or the shape of the relevant distributions. Individual sources of systematic uncertainty are considered to be uncorrelated. Correlations of a given systematic uncertainty with others are maintained across signal and background processes and channels. The following sections describe each of the systematic uncertainties considered in the analysis. Experimental uncertainties and background modelling uncertainties (Sects. 6.3.1, 6.3.2) are marginalised during the unfolding procedure, while signal modelling uncertainties, uncertainties due to Monte Carlo sample size, PDF uncertainties and unfolding response uncertainties (Sects. 6.3.3, 6.3.4) are added in quadrature to the unfolded uncertainty.

#### Experimental uncertainties

**Jet energy scale and resolution:** The jet energy scale (JES) and its uncertainty have been derived by combining information from test-beam data, LHC collision data and simulation [43]. The jet energy scale uncertainty is split into 22 uncorrelated components which can have different jet $p_{\text {T}}$ and $\eta$ dependencies and are treated independently in this analysis. The jet energy resolution (JER) has been determined as a function of jet $p_{\text {T}}$ and rapidity using dijet events from data and simulation. The JER in data and in simulation are found to agree within 10 %, and the corresponding uncertainty is assessed by smearing the jet $p_{\text {T}}$ in the simulation. The JES and JER uncertainties represent the leading sources of uncertainty associated with reconstructed objects in this analysis.

**Heavy- and light-flavour tagging:** The efficiencies to tag jets from *b*-quarks, *c*-quarks, and light quarks are measured in data as a function of $p_{\text {T}}$ (and $\eta$ for light-quark jets), and these efficiencies are used to adjust the simulation to match data. The uncertainties in the calibration are propagated through this analysis and represent a minor source of uncertainty.

**Jet reconstruction and identification:** The uncertainty associated with the jet reconstruction efficiency is assessed by randomly removing 0.2 % of the jets with $p_{\text {T}}$ below $30~\mathrm GeV{}$, to match the measured jet inefficiency in data for this $p_{\text {T}}$ range [43]. The uncertainty on the efficiency that each jet satisfies the JVF requirement is estimated by changing the JVF cut value from its nominal value by $\pm 0.1$, and repeating the analysis using the modified cut value. Both uncertainties have a negligible impact on the measurement.

**Leptons:** Uncertainties associated with leptons affect the reconstruction, identification and trigger efficiencies, as well as the lepton momentum scale and resolution. They are estimated from $Z\rightarrow \ell ^+\ell ^-$ ($\ell =e,\mu$), $J/\psi \rightarrow \ell ^+\ell ^-$ and $W\rightarrow e\nu$ processes using techniques described in Refs. [35, 36, 88]. The combined effect of all these uncertainties results in an overall normalisation uncertainty on the signal and background of approximately 1.5 %. Charge misidentification is not considered as it is small [88] and has a negligible impact on the measurement.

**Missing transverse momentum:** The $E_{\text {T}}^{\text {miss}}$ reconstruction is affected by uncertainties associated with leptons, jet energy scales and resolutions which are propagated to the $E_{\text {T}}^{\text {miss}}$ calculation. Additional small uncertainties associated with the modelling of the underlying event, in particular its impact on the $p_{\text {T}}$ scale and resolution of unclustered energy, are also taken into account. All uncertainties associated with the $E_{\text {T}}^{\text {miss}}$ have a negligible effect.

**Luminosity:** The uncertainty on the integrated luminosity is 2.8 %, affecting the overall normalisation of all processes estimated from MC simulation. It is derived following the methodology detailed in Ref. [32]. The impact of this uncertainty is negligible in this measurement.

#### Background modelling

***W*** **+jets**: The predictions of normalisation and flavour composition of the *W*+jets background are affected by large uncertainties, but the in situ data-driven technique described in Sect. 5.2 reduces these to a negligible level. All sources of uncertainty other than normalisation are propagated to the $W$+jets estimation.

***Z*** **+jets**: Uncertainties affecting the modelling of the *Z*+jets background include a 5 % normalisation uncertainty from the theoretical NNLO cross section [74], as well as an additional 24 % normalisation uncertainty added in quadrature for each additional inclusive jet-multiplicity bin, based on a comparison among different algorithms for merging LO matrix elements and parton showers [89]. The normalisation uncertainties for *Z*+jets are described by three uncorrelated nuisance parameters corresponding to the three *b*-tag multiplicities considered in the analysis.

**Multijet background:** Uncertainties on the multijet background estimated via the Matrix Method receive contributions from the size of the data sample as well as from the uncertainty on $\epsilon _{\mathrm {fake}}$, estimated in different control regions. A normalisation uncertainty of 50 % due to all these effects is assigned independently to the electron and muon channels and to each *b*-tag multiplicity, leading to a total of six uncorrelated uncertainties.

**Other physics backgrounds:** Uncertainties affecting the normalisation of the single-top-quark background include a +5 %/–4 % uncertainty on the total cross section estimated as a weighted average of the theoretical uncertainties on *t*-, *Wt*- and *s*-channel production [80–82]. Including an additional uncertainty in quadrature of 24 % per additional jet has a negligible impact on the measurement. Uncertainties on the diboson background normalisation include 5 % from the NLO theoretical cross sections [84] added in quadrature to an uncertainty of 24 % due to the extrapolation to the high jet-multiplicity region, following the procedure described for *Z*+jets.

#### Signal modelling

In order to investigate the impact of uncertainties on the $t\bar{t}$ signal modelling, additional samples generated with Powheg-Box interfaced to Herwig, Mc@nlo interfaced to Herwig and AcerMC interfaced to Pythia are considered (see Sect. 5.1 for more details). Different predictions and response matrices built with those $t\bar{t}$ samples are used to repeat the full analysis procedure isolating one effect at the time. For each case, the intrinsic asymmetry and the unfolded asymmetry are measured. The intrinsic asymmetry is the asymmetry generated in each Monte Carlo sample before the simulation of the detector response. Double differencees between the intrinsic (int) asymmetry and the unfolded (unf) values of the nominal (nom) and the alternative (alt) sample are considered as uncertainties to account for the different $A_{\text {C}}$ predictions of the different samples, $(A_{\text {C}}{}^{\mathrm {int, nom}} - A_{\text {C}}{}^{\mathrm {int, alt}}) - (A_{\text {C}}{}^{\mathrm {unf, nom}} - A_{\text {C}}{}^\mathrm {{unf, alt}})$. This is referred to as the double difference.

**NLO generator:** The uncertainty associated with the choice of NLO generator is estimated from the double difference of the parton-level $A_{\text {C}}$ and unfolded $A_{\text {C}}$ comparing Powheg-Box interfaced to Herwig (nom) and Mc@nlo interfaced to Herwig (alt).

**Fragmentation model:** The uncertainty associated with the fragmentation model is estimated from the double difference of the parton-level $A_{\text {C}}$ and unfolded $A_{\text {C}}$ comparing Powheg-Box interfaced to Pythia (nom) and Powheg-Box interfaced to Herwig (alt).

**Initial- and final-state radiation (ISR/FSR):** The uncertainty associated with the ISR/FSR modelling is estimated using the AcerMC generator where the parameters of the generation were varied to be compatible with the results of a measurement of $t\bar{t}$ production with a veto on additional central jet activity [90]. Two variations producing more and less ISR/FSR are considered. The uncertainty is estimated from half of the double difference of the parton-level $A_{\text {C}}$ and unfolded $A_{\text {C}}$ comparing Powheg-Box (nom) and AcerMC (alt) interfaced to Pythia producing more and less ISR/FSR.

#### Others

**Monte Carlo sample size:** To assess the effect on the measurement of the limited number of Monte Carlo events, an ensemble of 1000 response matrices, each of them fluctuated according to the raw number of simulated events, is produced. Unfolding is repeated with the same pseudo-dataset for each fluctuated response matrix. The uncertainty is estimated as the standard deviation of the ensemble of the 1000 $A_{\text {C}}$ values obtained. The estimated systematic uncertainty associated with limited number of Monte Carlo events is about ten times smaller than the data statistical uncertainty; this is consistent with the size of the available Monte Carlo sample.

**PDF uncertainties:** The choice of PDF in simulation has a significant impact on the charge asymmetry of the simulated $W$+jets background. Since this asymmetry is exploited to calibrate the $W$+jets prediction, the related uncertainty has to be estimated. The uncertainty on the PDFs is evaluated using three different PDF sets: CT10 [58], MSTW 2008 [91] and NNPDF2.1 [92]. For each set, the PDFs are varied based on the uncertainties along each of the PDF eigenvectors. Each variation is applied by reweighting the $W$+jets sample event-by-event. The $A_{\text {C}}$ measurements are repeated for each varied $W$+jets template and the uncertainty is estimated as half of the largest difference between any variation of CT10 and MSTW 2008, and the $\pm 1\sigma$ variations for NNPDF2.1. The resulting uncertainties are small, but non-negligible. The impact of uncertainties related to PDFs are found to be negligible in $t\bar{t}$ modelling.

**Unfolding response:** The response of the unfolding procedure, i.e. any non-linearity or bias, is determined using a set of six pseudo-datasets, each of them being composed of the default $t\bar{t}$ signal reweighted to simulate an asymmetry and the default MC simulation predictions. The injected $A_{\text {C}}$ value ranges between $-$0.2 and 0.2 depending on the differential variable and bin. The six reweighted pseudo-datasets are unfolded using the default response matrix and the uncertainty associated with the unfolding response is calculated as: $A_\mathrm {C}^{\mathrm {meas}} - (A_\mathrm {C}^{\mathrm {meas}}- b)/a$, with *a* and *b* the slope and offset of a linear fit of the generator-level (intrinsic) $A_{\text {C}}$ versus unfolded $A_{\text {C}}$ of the six reweighted pseudo-datasets previously defined and $A_\mathrm {C}^{\mathrm {meas}}$ the measured value in data.

**Fig. 2:**
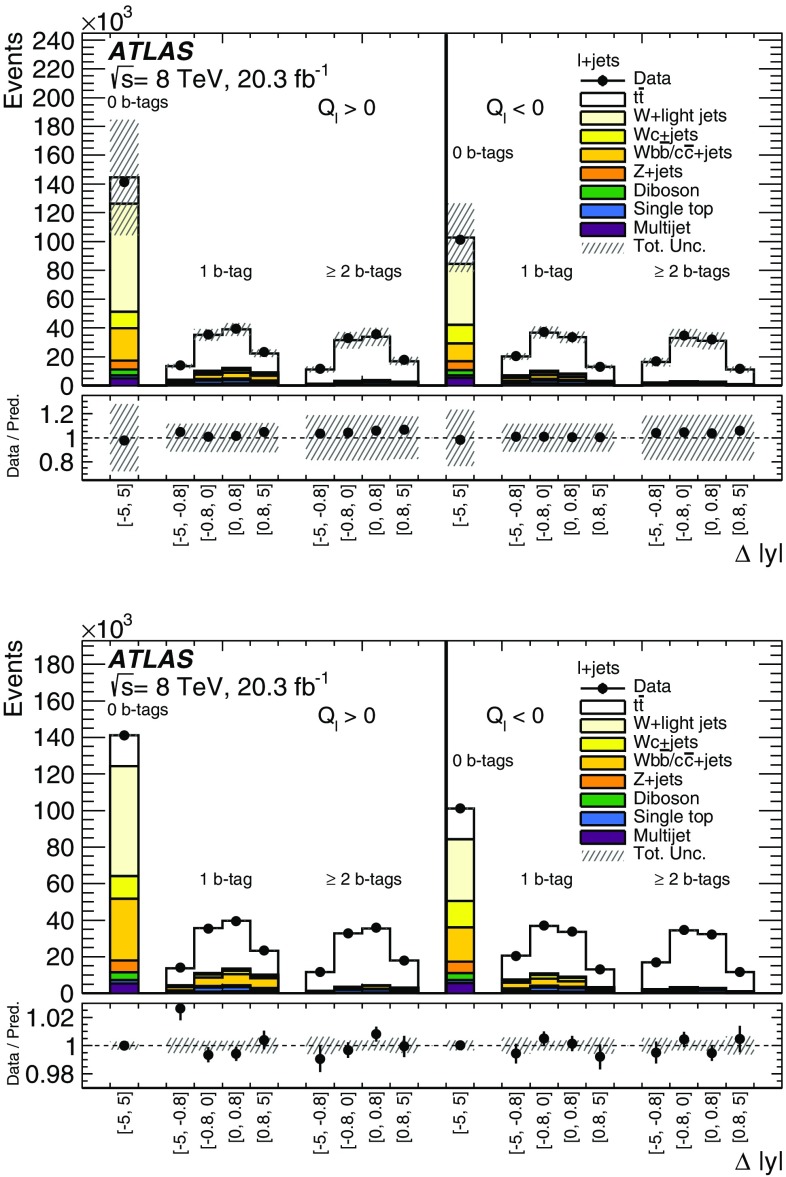
Comparison between prediction and data for the 18 bins used in the inclusive $A_{\text {C}}$ measurement before (*top*) and after (*bottom*) the simultaneous unfolding procedure and *W*+jets in situ background calibration, including only uncertainties that are marginalized. The $\Delta {}|y|$ distribution in four bins is considered for the $t\bar{t}$ -enriched event samples with exactly one and at least two *b*-jets; a single bin is considered for the background-enriched sample with zero *b*-jets. After the calibration, the background components are scaled to the measured values for the nuisance parameters, and the prediction for $t\bar{t}$ events in each bin is estimated by folding the measured parton-level parameters through the response matrix. The *bottom part of each plot* shows the ratio of the data to the predicted value together with combined statistical and systematic uncertainties

### Measurement

A fit is performed which maximises the extended likelihood of Eq. (6). In this fit, the events are further separated based on the sign of the lepton charge $Q_\ell$. The measurements are then performed using a combination of six channels based on the lepton charge ($Q_\ell >0$ and $Q_\ell <0$) and the *b*-jet multiplicity (zero *b*-jets, one *b*-jet, at least two *b*-jets). The $\Delta {}|y|$ distribution is split into four bins in all the channels except the zero *b*-jets channel, as no extra information for $A_{\text {C}}$ is expected. Four bins in $\Delta {}|y|$ are considered in each differential bin of all differential measurements.

The $W$+jets in situ calibration procedure consists of fitting the calibration factors $K_{b\bar{b} {}/c\bar{c} {}}$, $K_c$ and $K_{\text {light}}$ for scaling the flavor components of the $W$+jets background with different charge asymmetries, assuming uniform prior probabilities $\pi$ during the posterior probability estimation defined in Eq. (7). The *b*-jet multiplicity provides information about the heavy- and light-flavour composition of the $W$+jets background, while the lepton charge asymmetry is used to determine the normalisation of each component. Figure 2 shows the different $W$+jets contributions for the different *b*-jet multiplicities and lepton charges. In addition to the expected number of $t\bar{t}$ events for each bin in $\varvec{T}$, the $W$+jets calibration factors are free parameters in the likelihood. The posterior probability density is thus

7 $$\begin{aligned}&p\left( \varvec{T}{}|\{\varvec{D}{}_1\cdots {}\varvec{D}{}_{N_{\mathrm ch}}\}\right) \nonumber \\&\quad = \int \prod _{i=1}^{N_{\mathrm ch}}\mathcal {L}\left( \varvec{D}{}_i|\varvec{R}{}_i(\varvec{T}{};\varvec{\theta {}}{}_{\mathrm s}),\varvec{B}{}_i(K_{b\bar{b} {}/c\bar{c} {}},K_c,K_{\text {light}};\varvec{\theta {}}{}_{\mathrm s},\varvec{\theta {}}{}_{\mathrm b})\right) \nonumber \\&\qquad \times \mathcal {N}(\varvec{\theta {}}{}_{\mathrm s}) \mathcal {N}(\varvec{\theta {}}{}_{\mathrm b}) ~\pi {}(\varvec{T}{}) ~\pi {}(K_{b\bar{b} {}/c\bar{c} {}}) ~\pi {}(K_c) \\&\qquad \times ~\pi {}(K_{\text {light}}) ~\mathrm {d}\varvec{\theta {}}{}_{\mathrm s} ~\mathrm {d}\varvec{\theta {}}{}_{\mathrm b}, \end{aligned}$$

where $\varvec{B}{}=\varvec{B}{}(K_{b\bar{b} {}/c\bar{c} {}},K_c,K_{\text {light}};\varvec{\theta {}}_{\mathrm s},\varvec{\theta {}}{}_{\mathrm b})$ is the total background prediction, the probability densities $\pi {}$ are uniform priors and $\varvec{R}$ is the reconstructed signal prediction. Two categories of nuisance parameters are considered: the normalisation of the background processes ($\varvec{\theta {}}{}_{\mathrm b}$), and the uncertainties associated with the object identification, reconstruction and calibration ($\varvec{\theta {}}{}_{\mathrm s}$). While the first ones only affect the background predictions, the latter, referred to as object systematic uncertainties, affect both the reconstructed distribution for $t\bar{t}$ signal and the total background prediction. The $W$+jets calibration factors are found to be $K_{b\bar{b} {}/c\bar{c} {}}=1.50\pm 0.11$, $K_c = 1.07\pm 0.27$ and $K_{\text {light}} = 0.80\pm 0.04$, where the uncertainties include both the statistical and systematic components.

The final numbers of expected and observed data events after the full event selection, marginalisation of nuisance parameters and *W*+jets in situ calibration are listed in Table 1, while Fig. 2 shows the good level of agreement between the data and expectation before and after marginalisation for the six channels. In both cases, the uncertainties that are marginalized are shown. Since these uncertainties are correlated for the background and signal components, the total combined marginalized uncertainty is smaller than the sum of the constituent parts.

**Table 1:** Observed number of data events compared to the expected number of signal events and different background contributions for different *b*-tagging multiplicities in the combined $\mu {}$+jets and $e$+jets channels. These yields are shown after marginalisation of the nuisance parameters and the in situ calibration of the *W*+jets background, and the marginalized uncertainties are shown. The marginalized uncertainties for each background and signal component are correlated, and the correlation is taken into account in their combination

Channel	$\ell {}+\text {jets}$ 0-tag	$\ell {}+\text {jets}$ 1-tag	$\ell {}+\text {jets}$ 2-tag
Single top	$3400 \pm 400$	$12{,}100\pm 1300$	$8700\pm 900$
*W*+jets	$173{,}000\pm 9000$	$45{,}000\pm 4000$	$8600\pm 700$
*Z*+jets	$13{,}000\pm 6000$	$3900\pm 2000$	$1900\pm 900$
Diboson	$8000\pm 4000$	$2000\pm 900$	$400\pm 200$
Multijets	$10{,}800\pm 3500$	$6300\pm 2000$	$2200\pm 700$
Total background	$208{,}500\pm 1300$	$69{,}600\pm 2600$	$21{,}800\pm 1300$
$t\bar{t}$	$33{,}900\pm 1200$	$146{,}900\pm 2700$	$171{,}600\pm 1500$
Total expected	$242{,}400\pm 600$	$216{,}500\pm 500$	$193{,}400\pm 400$
Observed	242,420	216,465	193,418

## Results

### Inclusive measurement

The inclusive $t\bar{t}$ production charge asymmetry is measured to be

$$\begin{aligned} A_{\text {C}}{}= 0.009\pm 0.005~(\mathrm{{stat.}}+\mathrm{{syst.}}), \end{aligned}$$

compatible with the SM prediction, $A_{\text {C}}{}=0.0111\pm 0.0004$ [1].

Since the background estimation is part of the Bayesian inference procedure described in Sect. 6.2, it is not possible to study the impact of systematic uncertainties by repeating unfolding on data with varied templates, without using marginalisation. Instead, the expected impact of systematic uncertainties is studied with pseudo-data distributions corresponding to the sum of the background and signal predictions. For each source of uncertainty, the $\pm {}1\sigma$ variations of the predictions are used to build the pseudo-data, and the unfolding procedure is repeated. The baseline background templates and response matrices, as in the actual measurements, are used. Table 2 shows the average asymmetry variation $\delta {A_{\text {C}}{}}$ computed, for each source of uncertainty, as $|A_{\text {C}}{}(+1\sigma )-A_{\text {C}}{}(-1\sigma )|/2$, but only the uncertainties having a variation above 10 % of the statistical uncertainty are reported in the table. The total uncertainty associated with the marginalised systematic uncertainties is estimated by subtracting in quadrature the statistical term from the total marginalised uncertainty. It yields 0.002 (category (a) in Table 2). The total, non-marginalised uncertainty associated with systematic uncertainties is estimated by summing in quadrature sources from category (b) in Table 2.

**Table 2:** Impact of individual sources of uncertainty on the inclusive $A_{\text {C}}$ measurement. All uncertainties described in Sect. 6.3 are considered, but only the ones having a variation above $10\%$ of the statistical uncertainty are reported in the table. Systematic uncertainties in group (a) are marginalised while systematic uncertainties in group (b) are added in quadrature to the marginalised posterior

	Source of systematic uncertainty	$\delta {A_{\text {C}}{}}$
(a)	Jet energy scale and resolution	0.0016
	Multijet background normalisation	0.0005
(b)	Initial-/final-state radiation	0.0009
	Monte Carlo sample size	0.0010
	PDF	0.0007
	Statistical uncertainty	0.0044
	Total uncertainty	0.0049

The precision of the measurement is limited by the statistical uncertainty, and the main sources of systematic uncertainty are the signal modelling and the uncertainties with a large impact on the size of the $W$+jets background, such as the uncertainty on the jet energy scale and resolution.

### Differential measurements

**Fig. 3:**
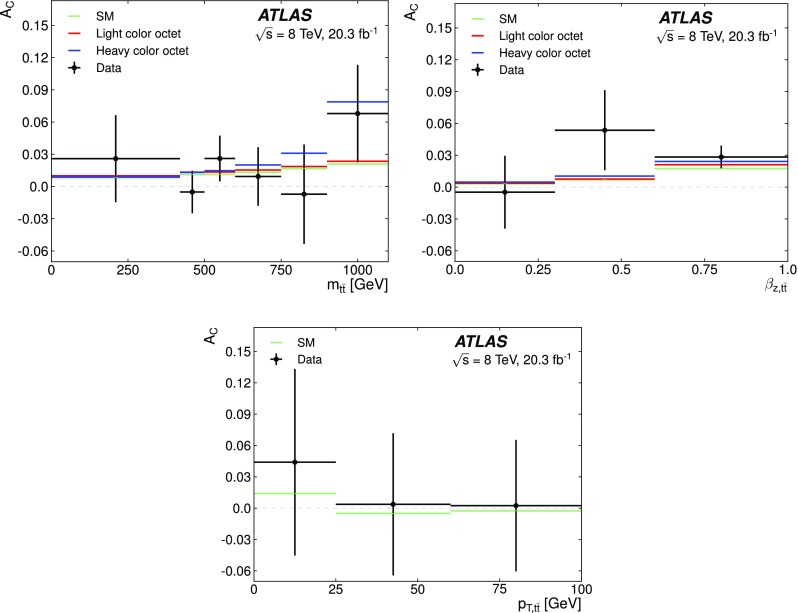
Measured $A_{\text {C}}$ values as a function of bin-averaged $m_{t\bar{t} {}}$, $\beta _{z,t\bar{t} {}}$ and $p_{\text {T},t\bar{t} {}}$, compared with predictions for SM [1] and for right-handed colour octets with masses below the $t\bar{t}$ threshold and beyond the kinematic reach of current LHC searches [93]. The BSM predictions are shown only for the *two top plots*. The bins are the same as the ones reported in Tables 3 and 4

The $A_{\text {C}}$ differential spectra are compared in Fig. 3 with the theoretical SM predictions, as well as with BSM predictions for right-handed colour octets with low and high masses [93]. The BSM predictions are not shown in the measurement as a function of $p_{\text {T},t\bar{t} {}}$ as they are LO $2\rightarrow 2$ calculations. The results are compatible with the SM, and it is not possible to distinguish between the SM and BSM models at this level of precision. The BSM models are tuned to be compatible with the Tevatron asymmetry measurements and the $A_{\text {C}}$ measurements at $\sqrt{s}=7$ TeV.

**Table 3:** Impact of individual sources of uncertainty on the measurement of $A_{\text {C}}{}$ in bins of $m_{t\bar{t} {}}{}$, $\beta _{z,t\bar{t} {}}{}$ and $p_{\text {T},t\bar{t} {}}{}$. All uncertainties described in Sect. 6.3 are considered, but only the ones having at least one bin with a variation above 10 % of the statistical uncertainty are reported in the table; the others are quoted as “–”. Systematic uncertainties in group (a) are marginalised while systematic uncertainties in group (b) are added in quadrature to the marginalised posterior

	Source of systematic uncertainty	$\delta A_{\mathrm C}$ in $m_{t\bar{t} {}}{}$ [GeV]					
		0–420	420–500	500–600	600–750	750–900	$>$900
(a)	Jet energy scale and resolution	0.010	0.007	0.007	0.009	0.013	0.009
	*b*-tagging/mis-tag efficiencies	0.006	0.005	0.005	0.005	0.008	0.005
	Missing transverse momentum	–	–	0.003	0.002	–	–
	Lepton reconstruction/identification	0.004	–	–	–	–	–
	Other backgrounds normalisation	0.009	0.006	–	0.002	–	–
(b)	Signal modelling	0.030	0.005	0.004	0.009	–	0.007
	Parton shower/hadronisation	–	0.005	–	–	0.010	0.011
	Initial-/final-state radiation	0.006	0.002	0.004	0.004	0.004	0.011
	Monte Carlo sample size	0.006	0.004	0.004	0.005	0.010	0.009
	PDF	0.004	0.002	0.002	0.004	0.005	0.007
	Statistical uncertainty	0.025	0.017	0.018	0.023	0.042	0.037
	Total	0.041	0.020	0.021	0.027	0.046	0.045

	Source of systematic uncertainty	$\delta A_{\mathrm C}$ in $\beta _{z,t\bar{t} {}}{}$					
		$<$0.3	0.3–0.6	0.6–1.0			
(a)	Jet energy scale and resolution	0.009	0.013	0.003			
	*b*-tagging/mis-tag efficiencies	0.003	0.003	0.001			
	Multijet background normalisation	0.003	–	–			
(b)	Signal modelling	0.025	0.027	0.002			
	Parton shower/hadronisation	0.009	0.010	0.006			
	Initial-/final-state radiation	0.006	–	–			
	Monte Carlo sample size	0.005	0.004	0.002			
	PDF	0.004	0.006	0.002			
	Statistical uncertainty	0.018	0.015	0.008			
	Total	0.034	0.038	0.011			

	Source of systematic uncertainty	$\delta A_{\mathrm C}$ in $p_{\text {T},t\bar{t} {}}{}$ [GeV]					
		0–25	25–60	$>$60			
(a)	Jet energy scale and resolution	0.009	0.009	0.003			
	Lepton energy scale and resolution	0.001	–	0.003			
	*b*-tagging/mis-tag efficiencies	0.007	0.008	0.003			
	Missing transverse momentum	0.002	0.004	0.002			
	Multijet background normalisation	0.005	0.003	$-$			
	Lepton reconstruction/identification	0.005	0.004	0.001			
	Other backgrounds normalisation	–	0.003	0.002			
(b)	Signal modelling	0.067	0.017	0.057			
	Parton shower/hadronisation	0.040	0.043	0.019			
	Initial-/final-state radiation	0.015	0.017	0.009			
	Monte Carlo sample size	0.006	0.008	0.003			
	PDF	0.009	0.009	0.004			
	Statistical uncertainty	0.017	0.028	0.014			
	Total	0.089	0.068	0.063			

Table 3 shows the average asymmetry variation $\delta {A_{\text {C}}{}}$ computed for each differential measurement, for each source of uncertainty, as explained in Sect. 7.1. The precision of the differential measurements is limited by the same factors as the inclusive result. The measurement versus $p_{\text {T},t\bar{t} {}}$ is particularly affected by the parton-shower model.

The resulting charge asymmetry $A_{\text {C}}$ is shown in Table 4 for the differential measurements as a function of $m_{t\bar{t} {}}$ $\beta _{z,t\bar{t} {}}$ and $p_{\text {T},t\bar{t} {}}$. The theoretical values are described in Ref. [1] (SM) and Ref. [93] (BSM), and they have been provided for the chosen bins. The correlation matrices are shown in Table 5 for the measurements as a function of $m_{t\bar{t} {}}$, $\beta _{z,t\bar{t} {}}$ and $p_{\text {T},t\bar{t} {}}$.

In regions with sensitivity to BSM (high values of $m_{t\bar{t} {}}$ and $\beta _{z,t\bar{t} {}}$), the uncertainty on the measurements is largely dominated by the available statistics, while in other regions the uncertainty on signal modeling and/or parton shower dominates.

**Table 4:** Measured charge asymmetry, $A_{\text {C}}$, values for the electron and muon channels combined after unfolding as a function of the $t\bar{t}$ invariant mass, $m_{t\bar{t} {}}$ (top), the $t\bar{t}$ velocity along the z-axis, $\beta _{z,t\bar{t} {}}$ (middle), and the $t\bar{t}$ transverse momentum, $p_{\text {T},t\bar{t} {}}$ (bottom). SM and BSM predictions, for right–handed colour octets with masses below the $t\bar{t}$ threshold (Light BSM) and beyond the kinematic reach of current LHC searches (Heavy BSM) [93], are also reported. The quoted uncertainties include statistical and systematic components after the marginalisation

	$$m_{t\bar{t} {}}{}$$ [GeV]					
$A_{\text {C}}{}$	$<420$	420–500	500–600	600–750	750–900	$>$900
Data	0.026 $\pm$ 0.041	$-0.005$ $\pm$ 0.020	0.026 $\pm$ 0.021	0.009 $\pm$ 0.027	$-0.007$ $\pm$ 0.046	0.068 $\pm$ 0.044
SM	0.0081$^{+0.0003}_{-0.0004}$	0.0112 $\pm$ 0.0005	0.0114$^{+0.0003}_{-0.0004}$	0.0134$^{+0.0003}_{-0.0005}$	0.0167$^{+0.0005}_{-0.0006}$	0.0210$^{+0.0003}_{-0.0002}$
Light BSM	0.0100 $\pm$ 0.0004	0.0134 $\pm$ 0.0006	0.0135$^{+0.0004}_{-0.0005}$	0.0155$^{+0.0005}_{-0.0006}$	0.0186$^{+0.0007}_{-0.0008}$	0.0235$^{+0.0006}_{-0.0005}$
Heavy BSM	0.0089 $\pm$ 0.0004	0.0132 $\pm$ 0.0006	0.0148$^{+0.0004}_{-0.0005}$	0.0201$^{+0.0004}_{-0.0006}$	0.0310$^{+0.0006}_{-0.0007}$	0.0788$^{+0.0007}_{-0.0006}$

	$$\beta _{z,t\bar{t} {}}{}$$					
$A_{\text {C}}{}$	$<$0.3	0.3–0.6	0.6–1.0			
Data	$-0.005$ $\pm$ 0.034	0.054 $\pm$ 0.038	0.028 $\pm$ 0.011			
SM	0.0031 $\pm$ 0.0003	0.0068 $^{+0.0002}_{-0.0003}$	0.0175 $^{+0.0007}_{-0.0008}$			
Light BSM	0.0037 $\pm$ 0.0004	0.0075 $\pm$ 0.0004	0.0211 $^{+0.0007}_{-0.0008}$			
Heavy BSM	0.0048 $\pm$ 0.0004	0.0103 $\pm$ 0.0004	0.0242 $^{+0.0007}_{-0.0008}$			

	$$p_{\text {T},t\bar{t} {}}{}$$ [GeV]					
$A_{\text {C}}{}$	$<$25	25–60	$>$60			
Data	0.044 $\pm$ 0.088	0.004 $\pm$ 0.066	0.002 $\pm$ 0.062			
SM	0.0141 $\pm$ 0.0007	$-0.0051$ $\pm$ 0.0003	$-0.0026$ $\pm$ 0.0002

**Table 5:** Correlation coefficients $\rho _{i,j}$ for the statistical and systematic uncertainties between the *i*-th and *j*-th bin of the differential $A_{\text {C}}$ measurement as a function of the $t\bar{t}$ invariant mass, $m_{t\bar{t} {}}$ (top), the $t\bar{t}$ velocity along the z-axis, $\beta _{z,t\bar{t} {}}$ (bottom left), and the transverse momentum, $p_{\text {T},t\bar{t} {}}$ (bottom right)

$\rho _{ij}$	$m_{t\bar{t} {}}{}$ [GeV]					
$m_{t\bar{t} {}}{}$ (GeV)	$<$420	420–500	500–600	600–750	750–900	$>$900
$<$420	1.	$-0.263$	0.076	$-0.034$	$-0.017$	$-0.001$
420–500		1.	$-0.578$	0.195	$-0.035$	$-0.002$
500–600			1.	$-0.591$	0.160	$-0.028$
600–750				1.	$-0.573$	0.132
750–900					1.	$-0.487$
$>$900						1.

$\rho _{ij}$	$\beta _{z,t\bar{t} {}}{}$					
$\beta _{z,t\bar{t} {}}{}$	$<$0.3	0.3–0.6	0.6–1.0			
$<$0.3	1.	$-0.262$	0.095			
0.3–0.6		1.	$-0.073$			
0.6–1.0			1.			

$\rho _{ij}$	$p_{\text {T},t\bar{t} {}}{}$ (GeV)					
$p_{\text {T},t\bar{t} {}}{}$ (GeV)	$<$25	25–60	$>$60			
$<$25	1.	$-0.812$	0.431			
25–60		1.	$-0.722$			
$>$60			1.			

### Interpretation

**Fig. 4:**
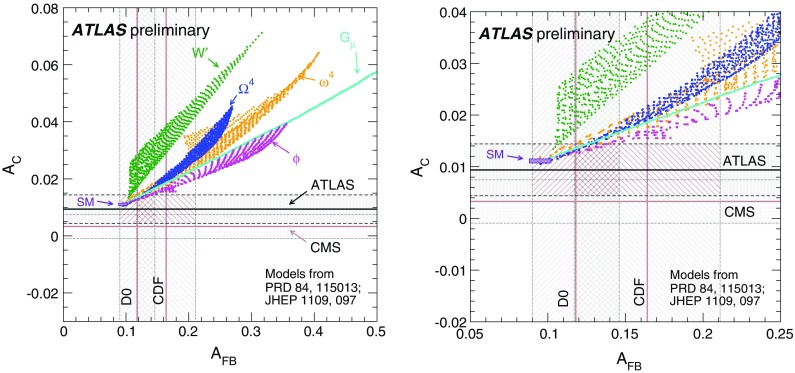
Measured inclusive charge asymmetries $A_{\text {C}}$ at the LHC versus forward–backward asymmetries $A_{\mathrm {FB}}$ at Tevatron, compared with the SM predictions [1, 9] as well as predictions incorporating various potential BSM contributions [20, 94]: a $W'$ boson, a heavy axigluon ($\mathcal {G}_{\mu }$), a scalar isodoublet ($\phi$), a colour-triplet scalar ($\omega ^4$), and a colour-sextet scalar ($\Omega ^4$). The *horizontal bands* and *lines* correspond to the ATLAS and CMS measurements, while the *vertical* ones correspond to the CDF and D0 measurements. The uncertainty bands correspond to a 68 % confidence level interval. The *figure on the right* is a zoomed-in version of the *figure on the left*

Figure 4 shows the inclusive $A_{\text {C}}$ measurement presented in Sect. 7. The measurement is compared to the $t\bar{t}$ forward–backward asymmetry^6^ $A_{\mathrm {FB}}$ measured at the Tevatron by CDF and D0 experiments. Predictions given by several BSM models, the details of which can be found in Refs. [20, 94], are also displayed. These BSM models include a $W'$ boson, a heavy axigluon ($\mathcal {G}_{\mu }$), a scalar isodoublet ($\phi$), a colour-triplet scalar ($\omega ^4$), and a colour-sextet scalar ($\Omega ^4$). For each model, the predictions for $A_{\mathrm {FB}}$ and $A_{\text {C}}$ are derived using the PROTOS generator [95] with the constraints described in Ref. [86]. The ranges of predicted values for $A_{\mathrm {FB}}$ and $A_{\text {C}}$ for a given set of BSM model are also shown. The BSM physics contributions are computed using the tree-level SM amplitude plus the one(s) from the new particle(s), to account for the interference between the two contributions. The phase-space of the parameters describing the various BSM models (such as the BSM particle masses and couplings) is limited by the measurement presented in this paper.

## Conclusion

The top-quark pair production charge asymmetry was measured with *pp* collisions at the LHC using an integrated luminosity of 20.3 $\text{ fb }^{-1}$ recorded by the ATLAS experiment at a centre-of-mass energy of $\sqrt{s}=8$ TeV in $t\bar{t}$ events with a single lepton (electron or muon), at least four jets and large missing transverse momentum. The reconstruction of $t\bar{t}$ events was performed using a kinematic fit. The reconstructed inclusive distribution of $\Delta {}|y|$ and the distributions as a function of $m_{t\bar{t} {}}$, $p_{\text {T},t\bar{t} {}}$ and $\beta _{z,t\bar{t} {}}$ were unfolded to obtain results that can be directly compared to theoretical computations. The measured inclusive $t\bar{t}$ production charge asymmetry is $A_{\text {C}}{} = 0.009 \pm 0.005$ (stat.$+$ syst.), to be compared to the SM prediction $A_{\text {C}}{}=0.0111\pm 0.0004$ [1]. All measurements presented in this paper are statistically limited and are found to be compatible with the SM prediction within the uncertainties. The precision of the measurements also allows for the exclusion of a large phase-space of the parameters describing various BSM models.

## Footnotes

1 ATLAS uses a right-handed coordinate system with its origin at the nominal interaction point (IP) in the centre of the detector and the *z*-axis coinciding with the axis of the beam pipe. The *x*-axis points from the IP to the centre of the LHC ring, and the *y*-axis points upward. Cylindrical coordinates (*r*,$\phi$) are used in the transverse plane, $\phi$ being the azimuthal angle around the beam pipe.

2 The pseudorapidity is defined in terms of the polar angle $\theta$ as $\eta = - \ln \tan (\theta /2)$ and transverse momentum and energy are defined relative to the beam line as $p_{\text {T}} {}= p \sin \theta$ and $E_{\mathrm T} =E \sin \theta$. The angular distances are given in terms of $\Delta R= \sqrt{(\Delta \eta )^2 + (\Delta \phi {})^2}$, where $\phi {}$ is the azimuthal angle around the beam pipe.

3 The method of selecting the PV is described in Sect. 4.

4 The jet vertex fraction is defined as the fraction of the total transverse momentum of the jet’s associated tracks that is contributed by tracks from the PV.

5
$m_{\mathrm T}^{W}= \sqrt{2 p^\ell _{\mathrm T} E_{\text {T}}^{\text {miss}} (1-\cos \Delta \phi )}$, where $p^\ell _{\mathrm T}$ is the transverse momentum (energy) of the muon (electron) and $\Delta \phi$ is the azimuthal angle separation between the lepton and the direction of the missing transverse momentum.

6 The $t\bar{t}$ asymmetry at the Tevatron is measured as a forward–backward asymmetry and defined as $A_{\mathrm {FB}}= \frac{N(\Delta y>0)-N(\Delta y<0)}{N(\Delta y>0)+N(\Delta y<0)}$.
